# PET Imaging of Self‐Assembled ^18^F‐Labelled Pd_2_L_4_ Metallacages for Anticancer Drug Delivery

**DOI:** 10.1002/chem.202202604

**Published:** 2022-11-23

**Authors:** Raúl Cosialls, Cristina Simó, Salvador Borrós, Vanessa Gómez‐Vallejo, Claudia Schmidt, Jordi Llop, Ana B. Cuenca, Angela Casini

**Affiliations:** ^1^ BISi-Bonds group Dept. of Organic and Pharmaceutical Chemistry Institut Químic de Sarrià, URL Vía Augusta 390 08017 Barcelona Spain; ^2^ CIC biomaGUNE Basque Research and Technology Alliance (BRTA) Paseo Miramón 182 20014 San Sebastián Gipuzkoa Spain; ^3^ Department of Organic and Inorganic Chemistry Universidad del País Vasco (UPV/EHU) Barrio Sarriena s/n 48940 Leioa Bizkaia Spain; ^4^ Grup d'Enginyeria de Materials (GEMAT) Institut Químic de Sarrià,URL Vía Augusta 390 08017 Barcelona Spain; ^5^ Chair of Medicinal and Bioinorganic chemistry Department of Chemistry Technical University of Munich Lichtenbergstr. 4 85748 Garching b. München Germany; ^6^ Centro de Innovación en Química Avanzada (ORFEO-CINQA) Spain; ^7^ Munich Data Science Institute Technical University of Munich 85748 Garching b. München Germany

**Keywords:** ammonium trifluoroborates, cisplatin, drug delivery, metallacages, PET imaging, self-assembly

## Abstract

To advance the design of self‐assembled metallosupramolecular architectures as new generation theranostic agents, the synthesis of ^18^F‐labelled [Pd_2_L_4_]^4+^ metallacages is reported. Different spectroscopic and bio‐analytical methods support the formation of the host‐guest cage‐cisplatin complex. The biodistribution profiles of one of the cages, alone or encapsulating cisplatin have been studied by PET/CT imaging in healthy mice in vivo, in combination to ICP‐MS ex vivo.

## Graphical abstract

**The synthesis of** two new self‐assembled ^18^F‐labelled [Pd_2_L_4_]^4+^ cages featuring a hydrophobic cavity able to encapsulate the anticancer drug cisplatin is reported, enabling biodistribution studies by positron emission tomography (PET) in vivo.

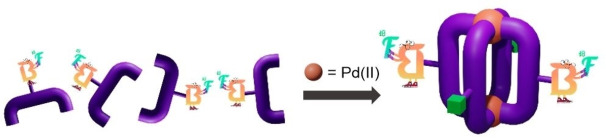

## Introduction

Supramolecular systems are attracting increasing attention in the development of nanomaterials for different applications.^[1]^ Self‐assembled porous metallacages are particularly attractive supramolecular coordination complexes featuring discrete molecular 3‐dimensional (3D)‐architectures with various appealing applications, including storage, separation, catalysis, recognition, as well as light emitting materials amongst others.^[2]^ In medicine, the biological properties of these well‐defined *molecular vessels* have recently been gaining momentum for drug delivery of therapeutics^[3]^ and imaging agents,^[4]^ as well as for the development of novel *theranostic* platforms.^[5]^ Among the different metal assemblies, some of us have focused on the advantages of a particular type of palladium‐based metallacage scaffold [Pd_2_L_4_]^4+^ (L=3,5‐bis(3‐ethynylpyridine)phenyl) as potential delivery system for the well‐established anticancer drug cisplatin.^[6]^ Thus, we developed the *exo*‐functionalization of the ligands to add different bioactive components, including fluorescent tags[^7^, ^8^, ^9^] and peptidic domains.^[10]^ It was also demonstrated that encapsulation of cisplatin in integrin targeted metallacages leads to reduced nephrotoxicity with respect to free cisplatin.^[11]^ Encapsulated cisplatin showed also higher in vitro cytotoxicity against cancer cells expressing the integrin receptors. ^[11]^ Noteworthy, [Pd_2_L_4_]^4+^ cages tethered to a blood brain barrier (BBB)‐translocating peptide and encapsulating radioactive pertechnetate were recently studied for their biodistribution in mouse models, and demonstrated the stability of the host‐guest (cage‐pertechnetate) complex and its brain penetration capability.^[12]^

While all the prominent proof‐of‐concept reports mentioned above can give a glimpse of a bright future for the use of metallacages as drug delivery systems, in vivo imaging studies of these supramolecular entities are still scarce.[^4^, ^13^] Most importantly, their design as novel theranostic platforms featuring both therapeutic and imaging modalities is still in its infancy. Besides, the ultimate question of the structural integrity of the metallacages upon in vivo injection remains unresolved. In this context, we considered that ^18^F‐labelling followed by in vivo Positron Emission Tomography (PET) imaging might offer interesting insights to that end. Additionally, and in contrast to optical imaging techniques, PET is fully translational into the clinical setting to assess whole body biodistribution. We considered the positron emitter fluorine‐18 (^18^F) as an appropriate radionuclide due to its wide availability and favorable properties (relatively long half‐life and short positron range).

## Results and Discussion

**Scheme 1:**
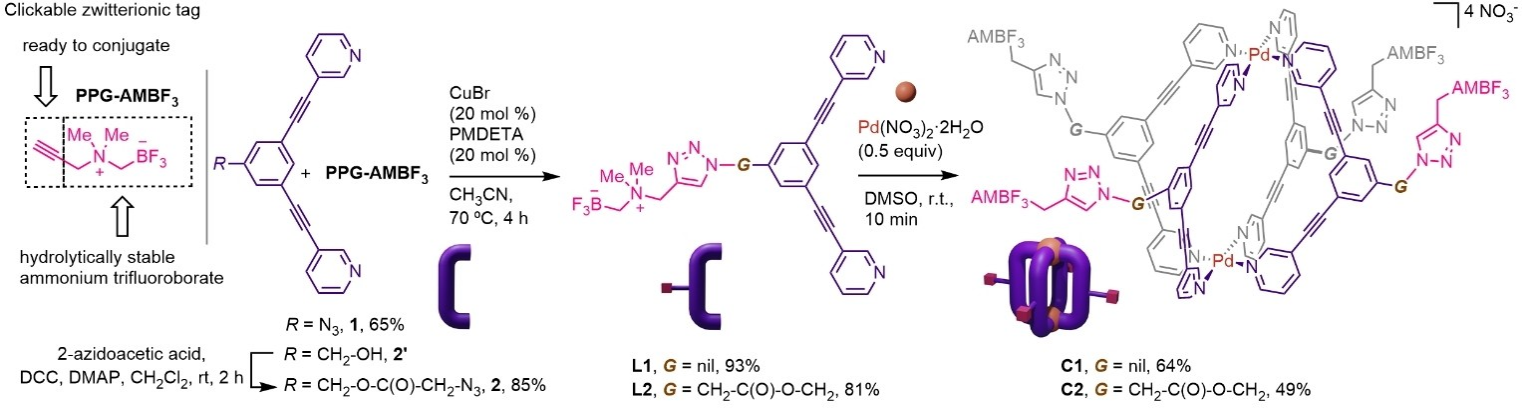
Structure of the clickable zwitterionic ammonium trifluoroborate (AMBF_3_) tag **PPG‐AMBF_3_**, and synthesis of ligands **L1** and **L2** and of the AMBF_3_‐modified cages **C1** and **C2**.

Among the possible ^18^F‐labelling strategies, our attention was drawn away from the common ^18^F‐carbon bond‐forming processes, focusing instead on the [^19^F]‐to‐[^18^F]‐boron isotopic exchange using ammonium trifluoroborate functionalities (AMBF_3_). These types of prosthetic groups, introduced by the Perrin laboratory, allow for efficient, single‐step, aqueous ^18^F‐labelling using as the labelling agent aqueous [^18^F]fluoride directly produced in the cyclotron, thus avoiding time‐consuming evaporation steps for solvent exchange.[^14a^, ^14b^] As an efficient route to append the AMBF_3_ fragment to the metallacage, we used a classical “click” approach, via a propargyl‐bearing AMBF_3_ reagent (**PPG‐AMBF_3_**,^[14c]^ Scheme 1) that could be conjugated to the azide‐modified cage ligand by Cu‐catalyzed alkyne‐azide cycloaddition reaction (CuAAC). Hence, two azide‐functionalized bis(pyridyl)ethynyl ligand precursors were initially considered, namely the derivative **1** ^[9a]^ with the azide attached directly to the central phenylene unit, and compound **2** in which a spacer is introduced between the ligand portion and the azide (see Figure S1a–b). The latter was prepared in an 85 % yield by Steglich‐type esterification of the benzylic alcohol *exo*‐functionalized ligand **2’** ^[15]^ with 2‐azido acetic acid. Next, the AMBF_3_‐modified ligands **L1** and **L2**, were synthesized through a CuAAC reaction of azides **1** and **2** with **PPG‐AMBF_3_** using the CuBr‐PMDETA (*N*,*N*,*N*′,*N*′′,*N*′′‐pentamethyldiethylenetriamine) as catalyst (Scheme 1).^16^ Data related to the NMR characterization of the ligands are reported in Figure S3–S4 in the Supporting Information.

With ligands **L1** and **L2** in hand, we proceeded to the self‐assembly of the homoleptic cages **C1** and **C2** with palladium di‐nitrate precursor which reached completion within 10 min after mixing (Scheme 1). The assembly of both [Pd_2_L_4_]^4+^ cages was unequivocally confirmed by ^1^H, DOSY NMR experiments, as well as by high‐resolution electrospray mass spectrometry (HR‐ESI‐MS) (Figure S5–S9 in the Supporting Information).^[6]^

**Figure 1:**
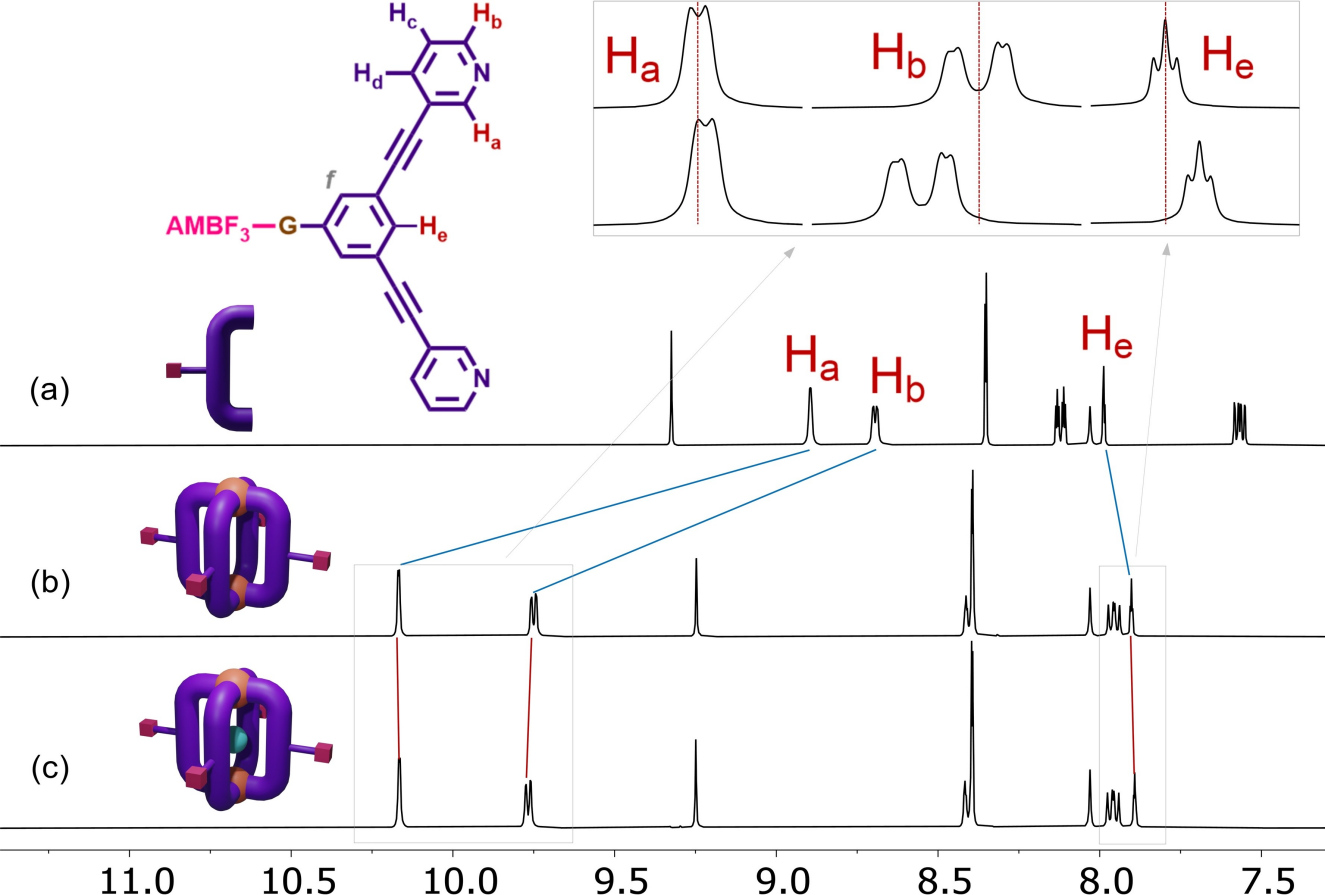
^1^H NMR spectrum (DMF‐*d_7_*, 298 K) of ligand **L1** (a), cage **C1**(NO_3_)_4_ (b) and (c) host‐guest adduct [**C1**(NO_3_)_4_⊃(cisplatin)].

Next, we verified that the newly assembled cages retained their ability to encapsulate cisplatin, an important feature for their potential as drug delivery systems. X‐ray diffraction analysis has shown that Pd_2_L_4_ cages can encapsulate up to 2 cisplatin molecules.[^3c^, ^6a^] It should be noted that the cage's 3,5‐bis(3‐ethynylpyridine)phenyl) scaffold creates a hydrophobic cavity whereby cisplatin encapsulation should be favoured over occupancy of the cavity by water molecules in solution.^[17]^ Thus, the host‐guest properties of **C1** and **C2** were studied by ^1^H and ^195^Pt NMR spectroscopy, as well as by HR‐ESI‐MS (Figure S10–S14). Metallacages **C1** or **C2** (1 equiv.) were dissolved in DMF‐*d*_7_ and then up to 3 equiv. of cisplatin were sequentially added stepwise, with each addition followed by a 10‐min sonication step. The ^1^H NMR spectrum recorded after the last addition revealed an identifiable downfield chemical shift of the *exo*‐facing proton H_b_ (Δ*δ*=0.02 ppm) for both cages, previously observed for similar cage systems upon cisplatin encapsulation.[^3c^, ^6b^] In addition, an observable upfield chemical shift (Δ*δ*=0.02 ppm) was found for the endohedral cavity‐facing H_e_, a plausible sign of the guest presence (Figure 1 and Figure S10). Afterwards, ^195^Pt NMR spectroscopy was also utilized to gain further insights into the cisplatin encapsulation in these cavities. Compared with free cisplatin, an upfield chemical shift of about −2 ppm was observed upon addition of 2 equiv. of cisplatin to a DMF‐*d_7_* solution of complex **C1** (Figure S12), in line with previous studies,^[9]^ and corroborating the idea of cisplatin encapsulation.

HR‐ESI‐MS analysis of [**C1**(NO_3_)_4_⊃cisplatin] and [**C2**(NO_3_)_4_⊃cisplatin], respectively, provided further evidence of the cage‐cisplatin encapsulation properties. For example, when a 1 : 2 mixture of **C1** and cisplatin in DMF was analysed, two clear peaks at *m/z*=839.8452 and *m/z*=1290.7616 appeared that could be unambiguously assigned to [Pd_2_(**L1**)_4_(NO_3_)_1_⊃cisplatin]^3+^ and [Pd_2_(**L1**)_4_(NO_3_)_2_⊃cisplatin]^2+^ host‐guest complexes, respectively (see Figure S13a–d). Moreover, a peak at *m/z*=1440.7548 was attributed to [**C1**(NO_3_)_2_⊃(cisplatin)_2_]^2+^ adducts. These species were also detected in 4 % DMSO in water. Analogous behaviour was observed for cage **C2** (see Figure S14a–c).

The encapsulation of cisplatin in **C1** was also studied by ^1^H DOSY NMR in DMF‐*d_7_*/D_2_O (90 : 10). Cisplatin alone in DMF shows a broad signal at ca. 4.2 ppm (H from NH_3_), with a diffusion coefficient ca. 6×10^−6^ m^2^/s.^11^ Upon addition of 2 equiv. cisplatin to 1 equiv. metallacage, the typical signal of free cisplatin disappeared in the DOSY plot (although marginally present in the ^1^H spectrum likely due to the fast exchange of free vs. encapsulated cisplatin species), while significant broadening of the cage signals were observed, accompanied by small shifts in their diffusion coefficients (Figure S15, spectrum *c*), suggesting that the cage cavity has been saturated to form the [**C1**⊃cisplatin] host‐guest complex.^11^ The cisplatin peak reappears only upon addition of a third equivalent of cisplatin to the sample (Figure S15, spectrum *d*), which should not undergo encapsulation.^[11]^ These data agree with the aforementioned HR‐ESI‐MS studies.

The feasibility of the ^18^F‐isotopic labelling of *exo*‐functionalized cages was then assessed. Initially, direct ^19^F‐to‐[^18^F] exchange (^18^F‐EX) from the preassembled **C1** and **C2** was attempted as a more straightforward approach. To this end, a wet no carrier added solution of ^18^F‐fluoride ion was generated and subsequently trapped on an anion exchange resin (QMA Cartridge). The ^18^F‐fluoride was next eluted with isotonic saline and then added to a vial containing unlabelled metallacages **C1** and **C2** in a DMF/water‐pyridazine⋅HCl buffer (pH=2) following previously established procedures.^[14a]^ The mixture was heated at 90 °C for 30–60 min. Unfortunately, the radio‐HPLC analysis of the ^18^F‐EX reaction evidenced major disassembly of the Pd^2+^ cages to the corresponding isotopically marked ^18^F‐**L1** and ^18^F‐**L2** ligands. Nevertheless, this experiment proved the efficient ^18^F‐EX reaction of the ligand framework prior to the self‐assembling process.

Given the fast kinetics of the assembly (few minutes), the final generation of the ^18^F‐labelled‐**C1** was straightforwardly achieved within the radioisotope half‐life time. Thus, ^18^F‐**L1** was prepared as above with slight modification of the experimental conditions (T=85 °C; reaction time=45 min, see Supporting Information for details). After confirmation of the quantitative formation of ^18^F‐**L1** by radio‐HPLC (Figure S21, chromatogram *a*), the labelled ^18^F‐**C1** cage was assembled by mixing ^18^F‐**L1** (2 equiv.) and 1 equiv. of Pd(NO_3_)_2_ ⋅ 2H_2_O in DMSO for 30 min in 95 % chromatographic yield (see Figure S21, chromatogram *b*). The same conditions were applied to achieve ^18^F‐**L2** and cage ^18^F‐**C2**. However, we decided to pursue the in vivo study only with **L1** since chromatographic yield of ligand ^18^F‐**L2** proved lower (40 %) than the corresponding ^18^F‐**L1**. Afterwards, we proceeded with encapsulation of cisplatin in the radio‐labelled cage. It should be noted that the radio‐HPLC retention time of the parent cage ^18^F‐**C1** and the same cage incubated for 5 min with 2 equiv. of cisplatin were virtually indistinguishable (Figure S21, chromatogram *c*). However, inductively coupled plasma optical emission spectrometry (ICP‐MS) analysis of the manually collected fractions of the latter sample did reveal presence of Pt for cisplatin loaded ^18^F‐**C1** (ca. 0.5 ng) but not in samples of free ^18^F‐**C1** and ^18^F‐**L1** (ca. 0.03 and 0.05 ng, respectively) used as controls; an observation that constitutes another indirect evidence of cisplatin encapsulation (Figure S22).

In parallel, the stability of cage **C1** over several hours was assessed through a series of ^1^H NMR experiments in different conditions that included 100 % DMSO‐*d_6_* and 4 % DMSO‐*d_6_* in D_2_O (Figure S16–S17). The stability of the [**C1**(NO_3_)_4_⊃cisplatin] complex was also monitored by ^1^H NMR over time in 4 % DMSO‐*d_6_* in D_2_O, and the results showed that the signals of the cage remain prominent only during the first hour (Figure S18). The origin of this enhanced cage instability in the presence of cisplatin is presently under investigation. Moreover, the stability of the free cage **C1** (0.15 mM injection concentration, 4 % DMSO in H_2_O) was also gauged by HR‐ESI‐MS. The resulting spectra showed the prominent presence of intact [**C1**(NO_3_)_n_]^z+^ species (Figure S19). Importantly, the presence of similar **C1**‐related species was also observed in 4 % DMSO in saline solution (up to 0.09 % NaCl) (Figure S20).

**Figure 2:**
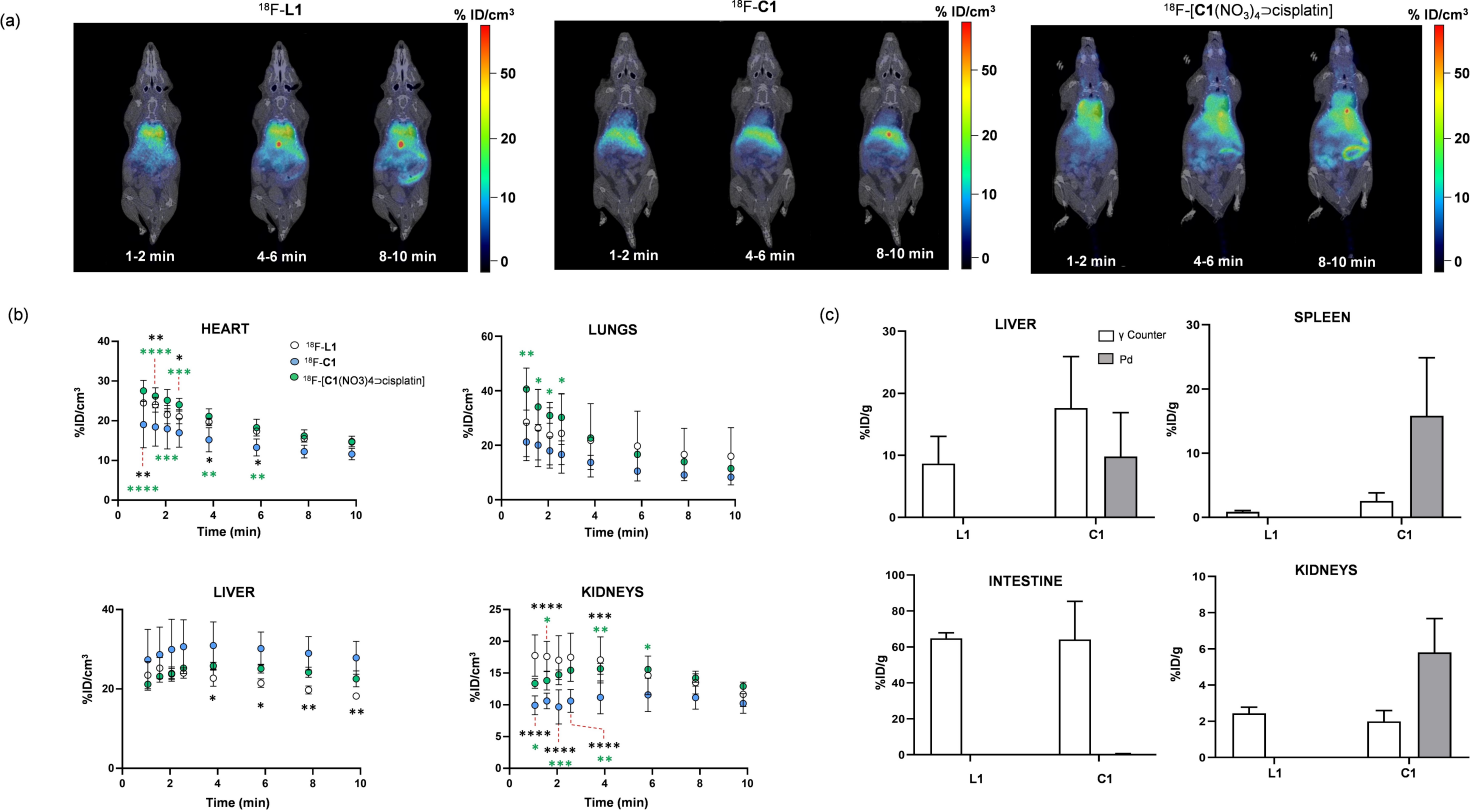
(a) Representative PET‐CT images (coronal projections of PET images co‐registered with representative CT slices) obtained at different time points after administration of ^18^F‐**L1**, ^18^F‐**C1** and ^18^F‐[**C1**(NO_3_)_4_⊃cisplatin]; (b) Time activity curves obtained from quantification of PET images. Values are expressed as percentage of injected dose per cubic centimetre of tissue (%ID/cm^3^). Values are expressed as mean ± standard deviation (n=4); probability values are depicted in black for ^18^F‐**L1** vs. ^18^F‐**C1** and in green for ^18^F‐**C1** vs. ^18^F‐[**C1**(NO_3_)_4_⊃cisplatin] as P<0.05, *; P<0.01, **, P<0.001, ***; and P<0.0001, ****; (c) Concentration of injected compound per gram of tissue as determined by dissection/gamma counting (white bars) and ICP‐MS (Pd; grey bars) after animal sacrifice; intestine represent only small intestine.

Next, biodistribution of ^18^F‐**L1**, ^18^F‐**C1** and cisplatin loaded ^18^F‐**C1** was investigated in healthy mice using PET imaging in combination with computed tomography (CT). PET acquisitions were started immediately after administration of labelled compounds and dynamic scans were acquired for 60 min. Quantification analysis of PET images were performed only in those organs clearly visualized on CT images (brain, heart, lungs, liver, kidneys, and bladder). Visual inspection of PET images obtained over the first 10 min after administration (Figure 2a) showed a very different profile for ^18^F‐**L1**, with presence of radioactivity in heart, lungs, intestines and gall bladder, with respect to cage ^18^F‐**C1**. The latter showed major accumulation in the liver and lower accumulation in the kidneys (both statistically significant in the time frame 0–4 min), as confirmed by image quantification (Figure 2b). Images also suggest lower accumulation of ^18^F‐**C1** in the gall bladder, although no quantification was carried out in this vesicle as, due to its small size, results could be subjected to severe partial volume effect. The observed differences in biodistribution provide evidence that the species arising upon injection of the supramolecular structure is distinct from the free ligand. In contrast, the biodistribution profile for ^18^F‐**L1** and ^18^F‐[**C1**(NO_3_)_4_⊃cisplatin] showed more similarities. This result suggests that a possible disassembly of the cisplatin loaded ^18^F‐**C1** may occur after in vivo injection, in accordance with the above‐mentioned NMR studies. Accumulation of radioactivity in the different organs reached similar values at longer time points, irrespectively of the administered compound (see Figure S23 for representative PET‐CT images). The lack of uptake in the bone confirms the absence of ^18^F‐defluorination of our ^19^F‐to‐[^18^F]‐trifluoroborate labelling strategy. After 60 min, ex vivo analysis based on dissection and gamma counting was carried out for ^18^F‐**L1** and ^18^F‐**C1** (Figure 2c). In this case, both compounds presented similar distribution pattern with major excretion through intestines (ca. 60 % of injected dose per gram of tissue ‐ %ID/g ‐ in the small intestine), further suggesting a possible non‐negligible disassembly of ^18^F‐**C1** to ^18^F‐**L1**. Analysis of the palladium levels in selected organs was performed by ICP‐MS after 60 min upon injection of ^18^F‐**C1** (Figure 2c) as another way of differentiating between the fates of the cage and the disassembled ligand. Noteworthy, Pd accumulation appears to be mostly uncoupled with respect to the ligand biodistribution. In fact, Pd was detected in spleen, kidney and liver, while being virtually absent in the intestine, where the ligand ^18^F‐**L1** prominently accumulates (Figure 2c). This result is in line with the observed different biodistribution among the ^18^F‐**L1** and ^18^F‐**C1**.

## Conclusion

In conclusion, we have reported on the straightforward synthesis and purification of metallacages as possible drug delivery systems and studied their biodistribution in vivo by PET imaging. The obtained results show that the species arising upon cages injection accumulates in different organs with respect to their ligands in the early time points. Moreover, cisplatin encapsulation seems to favour cage disassembly in vivo, as suggested by PET. Certainly, further optimization of the ligand system, for example using more electron‐donating tripyridyl ligands,^[18]^ is necessary to enable kinetically robust cage complexes. Alternatively, the use of Pt(II) ions instead of Pd(II) could also increase the kinetic stability, although the self‐assembly may require different reaction conditions not necessarily compatible with the radiolabelling procedure.^19^ The cisplatin encapsulation process also requires in‐depth investigation and should be performed on targeted and more hydrophilic cage systems. Overall, owing to their unique physicochemical properties, metal‐coordinated supramolecular self‐assemblies, including the selected metallacages, can bridge the boundary between traditional inorganic and organic materials, and our work further progresses their design for biomedical applications.

## Experimental Section

**Materials and methods**: All commercially acquired reagents were used as received unless indicated otherwise. 2‐azidoacetic acid,^[20]^ 3,3′‐((5‐azido‐1,3‐phenylene)bis(ethyne‐2,1‐diyl))dipyridine (**1**),^[9a]^ (3,5‐bis(pyridin‐3‐ylethynyl)phenyl)methanol (**2’**)^[6b]^ and ((dimethyl(prop‐2‐yn‐1‐yl)ammonio)methyl)trifluoroborate (**PPG‐AMBF_3_**)^[14c]^ were prepared according to literature procedures or with slight modifications. HPLC grade ethanol, methanol and acetonitrile were purchased from Scharlab (Sentmenat, Barcelona, Spain). Reactions requiring inert atmosphere were conducted under argon atmosphere using standard Schlenk line techniques. Thin layer chromatography (TLC) was performed using Merck plastic‐backed plates of TLC Silica gel 60 F254; the plates were revealed using UV light at 254 nm or by staining using potassium permanganate. Standard Flash Column chromatography was accomplished using Merck silica gel (60 Å pore size, 70–230 μm mesh size). Automated Flash Column chromatography was performed by a Teledyne ISCO CombiFlash Rf200 system through pre‐packed RediSep Rf silica gel columns. HRMS data were acquired on a X500B SCIEX QTOF high‐resolution mass spectrometer (ESI mode). Spectroscopic experiments for the characterization of compounds and encapsulation studies were carried out at the Structural Determination facility of IQS on a Varian 400 NMR spectrometer (400 MHz for ^1^H, 100.5 MHz for ^13^C, 376 MHz for ^19^F and 128 MHz for ^11^B). ^195^Pt and ^1^H DOSY experiments were performed at the NMR unit of Universitat de Barcelona on a Bruker Avance III 400 MHz spectrometer and at TUM on a Bruker Avance III 500 MHz spectrometer. Chemical shifts (δ_Η_) are quoted in parts per million (ppm) and referenced to the appropriate NMR resonance, which for ^1^H measurements would correspond to the residual portion component of the deuterated solvent. The ^19^F and ^11^B chemical shift are referenced relative to CFCl_3_ and BF_3_⋅Et_2_O resonance at 0.00 ppm, respectively. The ^195^Pt chemical shift was referenced using an external reference of K_2_PtCl_4_ in D_2_O (−1610 ppm). Spin‐spin coupling constants (J) are reported in Hertz (Hz). Infrared spectra were recorded on a Thermo Scientific Nicolet iS10 FTIR spectrophotometer equipped with Smart iTR window and are reported in cm^−1^. Mediterranean C18 column (4.6×150 mm, 5 μm) as stationary phase and 0.1 % TFA water/acetonitrile (0 min 10 % acetonitrile; 0–2 min 20 % acetonitrile; 2–10 min 70 % acetonitrile; 10–14 min 70 % acetonitrile; 14–16 min 10 % acetonitrile; 16–20 min 10 % acetonitrile) as mobile phase at a flow rate of 1 mL/min and wavelength of 254 nm. ICP‐MS measurements were performed on a Thermo iCAP Q ICP‐MS instrument.

**Synthesis of azide precursor (2)**: (3,5‐bis(pyridin‐3‐ylethynyl)phenyl)methanol (**2’**) (400 mg, 1.3 mmol, 1 equiv.), 2‐azidoacetic acid (0.15 mL, 2.0 mmol, 1.5 equiv.) and 4‐dimethylaminopyridine (DMAP) (31 mg, 0.26 mmol, 0.2 equiv.) were charged into an oven‐dried 25 mL Schlenk tube and dissolved in anhydrous CH_2_Cl_2_ (15 mL). Then, *N*,*N*′‐dicyclohexylcarbodiimide (DCC) (425 mg, 2.0 mmol, 1.5 equiv.) was added to the tube and the mixture was allowed to stir at room temperature for 2 h. At this point, the reaction mixture was filtered and evaporated to dryness. The product was isolated using automated flash‐chromatography eluting with 1 : 0 to 0 : 1 cyclohexane:AcOEt gradient mixture (R_f_=0.3 in AcOEt). Pale orange powder, 429 mg, 85 %. 
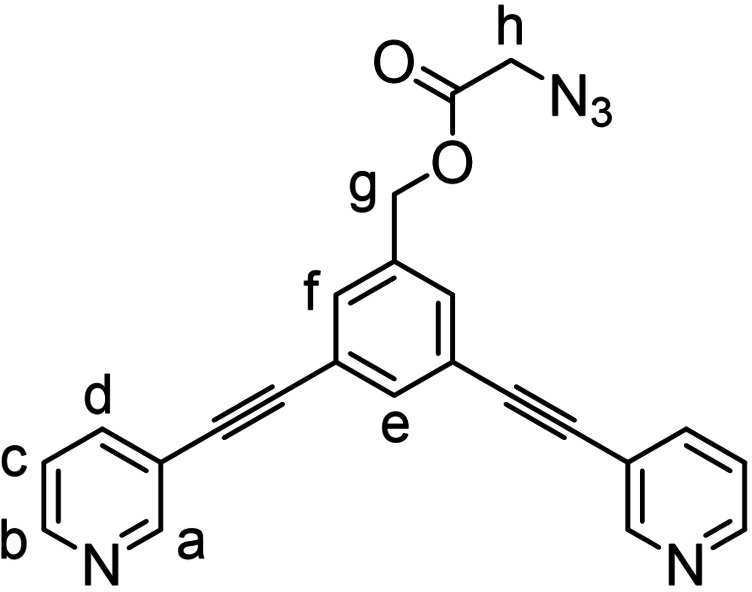


^1^H NMR (DMSO‐*d_6_*, 400 MHz) δ 8.79 (dd, *J*=2.2, 0.9 Hz, 2H, H_a_), 8.62 (dd, *J*=4.9, 1.7 Hz, 2H, H_b_), 8.01 (ddd, *J*=7.9, 2.2, 1.7 Hz, 2H, H_d_), 7.80 (t, *J*=1.6 Hz, 1H, H_e_), 7.70 (dd, *J*=1.5, 0.7 Hz, 2H, H_f_), 7.49 (ddd, *J*=7.9, 4.9, 0.9 Hz, 2H, H_c_), 5.26 (s, 2H, H_g_), 4.25 (s, 2H, H_h_). ^13^C NMR (DMSO‐*d_6_*, 100 MHz) δ 168.7, 151.7, 149.4, 138.7, 137.3, 133.8, 131.4, 123.7, 122.6, 118.9, 90.8, 87.3, 65.3, 49.5. FTIR (KBr) cm^−1^: 3032 (ar C−H st), 2930 (C−H st), 2108 (N_3_ st), 1749 (C=O st), 1595, 1478, 1407, 1286, 1186, 1023, 805, 704. HRMS‐ESI: calc. for C_23_H_16_N_5_O_2_ [M+H]^+^: m/z=394.1299; found 394.1286.

### Synthesis of ligands L1 and L2

*General procedure A*: The corresponding azide‐functionalized bis(pyridyl)ethynyl substrate (0.62 mmol, 1.5 equiv.) and ((dimethyl(prop‐2‐yn‐1‐yl)ammonio)methyl)trifluoroborate (**PPG‐AMBF_3_**) (68 mg, 0.41 mmol, 1 equiv.) were charged into an oven‐dried 25 mL Schlenk tube and dissolved in anhydrous CH_3_CN (19 mL). Then, a solution of CuBr (12 mg, 0.083 mmol, 0.2 equiv.) and *N*,*N*,*N*’,*N*’’,*N*’’‐pentamethyldiethylenetriamine (PMDETA) (17 μL, 0.083 mmol, 0.2 equiv.) in anhydrous CH_3_CN (1 mL), previously bubbled with argon for 15 min, was added. The mixture was allowed to stir at 70 °C for 4 h. After cooling to room temperature, the mixture was evaporated to dryness and the product was isolated using automatic flash‐chromatography with CH_2_Cl_2_:MeOH mixtures as indicated. 
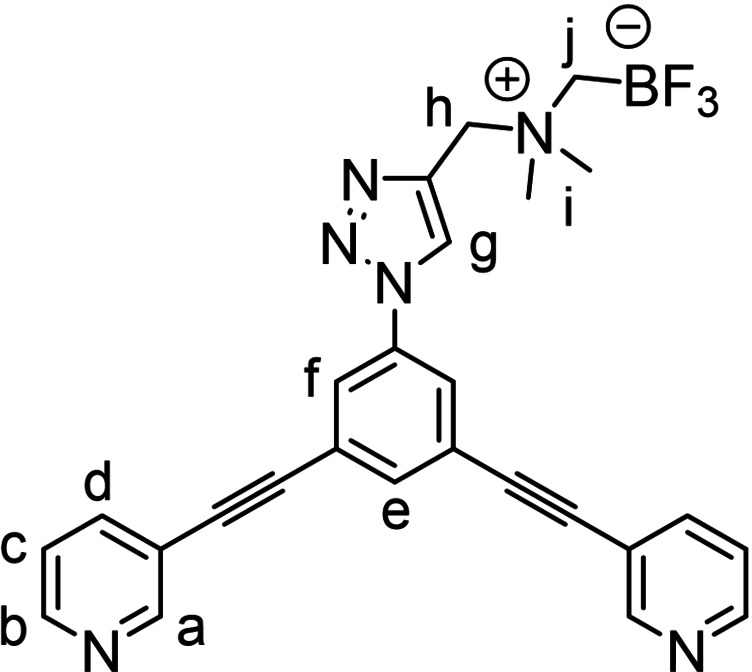


(**L1**) Prepared following the *General procedure A* from 3,3′‐((5‐azido‐1,3‐phenylene)bis(ethyne‐2,1‐diyl))dipyridine (**1**) and purified using automatic flash‐chromatography eluting with 1:0 to 95 : 5 CH_2_Cl_2_:MeOH gradient mixture (R_f_=0.4 in CH_2_Cl_2_:MeOH 9 : 1). Pale orange powder, 178 mg, 93 % yield. ^1^H NMR (DMSO‐*d_6_*, 400 MHz) δ 9.21 (s, 1H, H_g_), 8.83 (dd, *J*=2.2, 0.9 Hz, 2H, H_a_), 8.65 (dd, *J*=4.8, 1.7 Hz, 2H, H_b_), 8.28 (d, *J*=1.4 Hz, 2H, H_f_), 8.05 (ddd, *J*=7.9, 2.2, 1.7 Hz, 2H, H_d_), 7.96 (t, *J*=1.4 Hz, 1H, H_e_), 7.52 (ddd, *J*=7.9, 4.9, 0.9 Hz, 2H, H_c_), 4.65 (s, 2H, H_h_), 3.01 (s, 6H, H_i_), 2.37 (q, *J*_*H‐F*_=4.8 Hz, 2H, H_j_). ^13^C NMR (DMSO‐*d_6_*, 100 MHz) δ 151.8, 149.6, 138.8, 137.6, 137.0, 134.1, 126.6, 124.0, 123.8, 123.3, 118.7, 90.0, 88.4, 60.2, 51.7. ^19^F NMR (DMSO‐*d_6_*, 376 MHz) δ −135.1. ^11^B NMR (DMSO‐*d_6_*, 128 MHz) δ 1.93. ^1^H DOSY NMR (400 MHz, DMF‐*d_7_*): *D*=4.75×10^−6^ cm^2^/seg. FTIR (ATR) cm^−1^: 3118, 3026, 1590, 1475, 1413, 1036, 987, 967, 899, 889, 805, 700. HRMS‐ESI: calc. for C_26_H_23_BF_3_N_6_ [M+H]^+^: m/z=487.2024; found 487.2015. 
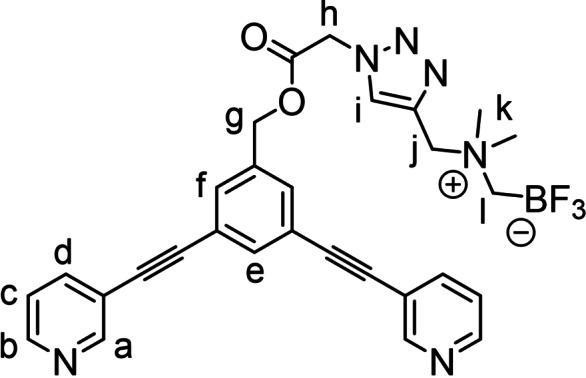


(**L2**) Prepared following the *General procedure A* from 3,5‐bis(pyridin‐3‐ylethynyl)benzyl 2‐azidoacetate (**2**) and purified using automatic flash‐chromatography eluting with 1:0 to 95 : 5 CH_2_Cl_2_:MeOH gradient mixture (R_f_=0.2 in CH_2_Cl_2_ MeOH 9 : 1). Pale orange powder, 187 mg, 81 % yield.

^1^H NMR (DMSO‐*d_6_*, 400 MHz) δ 8.80 (dd, *J*=2.2, 0.9 Hz, 2H, H_a_), 8.63 (dd, *J*=4.9, 1.7 Hz, 2H, H_b_), 8.42 (s, 1H, H_i_), 8.02 (ddd, *J*=7.9, 2.2, 1.7 Hz, 2H, H_d_), 7.81 (t, *J*=1.6 Hz, 1H, H_e_), 7.70 (dd, *J*=1.4, 0.7 Hz, 2H, H_f_), 7.50 (ddd, *J*=7.9, 4.9, 0.9 Hz, 2H, H_c_), 5.63 (s, 2H, H_h_), 5.28 (s, 2H, H_g_), 4.56 (s, 2H, H_j_), 2.93 (s, 6H, H_k_), 2.22 (q, *J*_*H‐F*_=4.7 Hz, 2H, H_l_). ^13^C NMR (DMSO‐*d_6_*, 100 MHz) δ 167.1, 151.7, 149.3, 138.7, 137.11, 136.5, 133.9, 131.4, 129.6, 123.9, 122.6, 119.2, 90.8, 87.4, 65.7, 59.9, 51.8, 50.6. ^19^F NMR (DMSO‐*d_6_*, 376 MHz) δ −135.3. ^11^B NMR (DMSO‐*d_6_*, 128 MHz) δ 1.87. FTIR (ATR) cm^−1^: 3148, 3030, 2956 (C−H st), 1752 (C=O st), 1596, 1477, 1407, 1199, 1022, 992, 969, 897, 804, 702. HRMS‐ESI: calc. for C_29_H_27_BF_3_N_6_O_2_ [M+H]^+^: m/z=559.2235; found 559.2210.

### Synthesis of metallacages C1 and C2

*General procedure B*: The corresponding AMBF_3_‐containing ligand (0.1 mmol, 2 equiv.) and Pd(NO_3_)_2_ ⋅ 2H_2_O (12 mg, 0.05 mmol, 1 equiv.) were charged into a 50 mL conical centrifuge tube and dissolved in DMSO (4 mL). The mixture was allowed to stir at room temperature for 1 h. At this point, the product was precipitated by addition of acetone (4 mL) and diethyl ether (40 mL). The mixture was centrifugated, decanted and the obtained solid was washed with diethyl ether (3 × 5 mL). The product was dried under high vacuum.

(**C1**) Prepared following the *General procedure B* from **L1**. Yellow powder, 40 mg, 64 %. ^1^H NMR (DMSO‐*d_6_*, 400 MHz) δ 9.81 (d, *J*=2.0 Hz, 2H, H_a_), 9.46 (dd, *J*=5.9, 1.4 Hz, 2H, H_b_), 9.17 (s, 1H, H_g_), 8.37 (d, *J*=1.4 Hz, 2H, H_f_), 8.31 (dt, *J*=8.1, 1.5 Hz, 2H, H_d_), 8.02 (t, *J*=1.4 Hz, 1H, H_e_), 7.87 (dd, *J*=8.0, 5.8 Hz, 2H, H_c_), 4.62 (s, 2H, H_h_), 2.97 (s, 6H, H_i_), 2.32 (q, *J*_*H‐F*_=4.7 Hz, 2H, H_j_). ^13^C NMR (DMSO‐*d_6_*, 100 MHz) δ 153.1, 151.0, 143.0, 137.7, 137.3, 133.9, 127.5, 126.6, 124.5, 123.3, 121.9, 92.5, 86.4, 60.0, 51.7. ^19^F NMR (DMSO‐*d_6_*, 376 MHz) δ −135.1. ^11^B NMR (DMSO‐*d_6_*, 128 MHz) δ 6.94. ^1^H DOSY NMR (400 MHz, DMF‐*d_7_*): *D*=2.1×410^−6^ cm^2^/seg. FTIR (ATR) cm^−1^: 3070, 1589, 1477, 1325, 1195, 1012, 882, 815, 695. HRMS‐ESI: calc. for [Pd_2_L_4_(NO_3_)_1_]^3+^: m/z=739.8558; found 739.8604.

(**C2**) Prepared following the *General procedure B* from **L2**. Orange powder, 33 mg, 49 %. ^1^H NMR (DMSO‐*d_6_*, 400 MHz) δ 9.74 (d, *J*=2.1 Hz, 2H, H_a_), 9.42 (dd, *J*=5.9, 1.4 Hz, 2H, H_b_), 8.38 (s, 1H, H_i_), 8.27 (dt, *J*=8.0, 1.5 Hz, 2H, H_d_), 7.90 (t, *J*=1.5 Hz, 1H, H_e_), 7.84 (dd, *J*=8.0, 5.8 Hz, 2H, H_c_), 7.76 (d, *J*=1.5 Hz, 2H, H_f_), 5.57 (s, 2H, H_h_), 5.26 (s, 2H, H_g_), 4.53 (s, 2H, H_j_), 2.91 (s, 6H, H_k_), 2.18 (q, *J*_*H‐F*_=4.8 Hz, 2H, H_l_). ^13^C NMR (DMSO‐*d_6_*, 100 MHz) δ 167.0, 152.9, 150.7, 142.9, 137.6, 136.5, 133.8, 132.6, 129.5, 127.4, 122.1, 122.0, 93.3, 85.4, 65.5, 59.8, 51.9, 50.5. ^19^F NMR (DMSO‐*d_6_*, 376 MHz) δ −135.3. ^11^B NMR (DMSO‐*d_6_*, 128 MHz) δ 7.01. FTIR (ATR) cm^−1^: 3067, 1753, 1479, 1332, 1196, 1023, 1007, 970, 897, 818, 695. HRMS‐ESI: calc. for [Pd_2_L_4_(NO_3_)_1_]^3+^: m/z=835.8887; found 835.8833.

**Cisplatin encapsulation and stability experiments**: Experimental procedures for the encapsulation studies of **C1** and **C2** by ^1^H NMR, ^195^Pt NMR, DOSY NMR and HR‐ESI‐MS, along with **C1** stability experiments by ^1^H NMR and HR‐ESI‐MS are provided in the Supporting Information.

### Radiolabelling

*Radiolabeling of* ^*18*^ *F‐**L1***: **L1** was labelled with fluorine‐18 by isotopic exchange reaction. Briefly, no‐carrier‐added [^18^F]F^−^ was obtained in 500 μL of ^18^O‐enriched water by proton irradiation. **L1** (200 μg) was suspended in 20 μL of DMF‐HCl‐pyridazine buffer (pH 2.0) and mixed with 20 μL of [^18^F]F^−^ (ca. 145±40 MBq). The reaction mixture was heated at 85 °C for 45 min. Afterwards, the crude was quenched with 2 mL of 5 % NH4OH solution and purified by preconditioned C18 cartridge (Sep‐Pak® Light, Waters) to selectively retain ^18^F‐**L1**. The desired product was eluted with 1 mL of EtOH, evaporated at 80 °C under N_2_ flow and re‐suspended in 1 mL of EtOH/saline (1 : 9) for subsequent in vivo injections. Quality control of ^18^F‐**L1** was performed via analytical radio‐HPLC (tr=7.5 min, Figure S21, chromatogram *a*). Total synthesis time: 1 h and 15 min.

*Formation of* ^*18*^ *F‐**C1***: Prepared following the procedure described above for non‐labelled **C1**, with minor modifications. To a vial containing evaporated ^18^F‐**L1**, 40 μL of Pd(NO_3_)_2_ ⋅ 2H_2_O in DMSO (1 mg/mL, 0.15 μmol, 1 equiv.) were added and the mixture was stirred for 30 min at room temperature. Quality control was performed via analytical radio‐HPLC (tr=8.6 min, Figure S21, chromatogram *b*). Then, the resulting solution was diluted with 1 mL of saline for in vivo injections.

*Encapsulation of cisplatin in* ^*18*^ *F‐**C1***: Prepared following the procedure described above. To a vial containing evaporated ^18^F‐**L1**, 40 μL of Pd(NO_3_)_2_ ⋅ 2H_2_O in DMSO (1 mg/mL, 0.15 μmol, 1 equiv.) were added and the mixture was stirred for 30 min at room temperature. The resulting ^18^F‐**C1** was added to a pre‐loaded Eppendorf containing 800 μL of cisplatin in ultrapure water (62.5 μg/mL, 2 equiv.). The reaction mixture was incubated during 5 min. Quality control was performed via analytical radio‐HPLC (tr=8.6 min, Figure S21, chromatogram *c*). Afterwards, 160 μL of saline were added to reach a final volume of 1 mL for in vivo injections.

### *In vivo* and *ex vivo* biodistribution studies

*Animals*: Female mice (BALB/cJRj, 8 weeks, Janvier; 9 animals) weighing 22±2 g were used to conduct the biodistribution studies. The animals were maintained and handled in accordance with the Guidelines for Accommodation and Care of Animals (European Convention for the Protection of Vertebrate Animals Used for Experimental and Other Scientific Purposes) and internal guidelines. All experimental procedures were approved by the internal committee and the local authorities.

*Biodistribution studies*: Mice were anesthetized by inhalation of 3 % isoflurane in pure O_2_ and maintained by 1.5–2 % isoflurane in 100 % O_2_. With the animal under anesthesia, ^18^F‐**L1**, ^18^F‐**C1** or cisplatin loaded ^18^F‐**C1** were injected intravenously via one of the lateral tail's veins (110 μL, 1.48±0.74 MBq, n=3 per compound). Dynamic whole body 60‐min PET scans were started immediately after administration using MOLECUBES β‐CUBE (PET) scanner. After each PET scan, whole‐body high‐resolution CT acquisitions were performed on the MOLECUBES X–CUBE (CT) scanner to provide anatomical information of each animal as well as the attenuation map for later image reconstruction. Random and scatter corrections were automatically applied during image reconstruction (3D OSEM reconstruction algorithm). PET‐CT images of the same mouse were co‐registered and analyzed using the PMOD image processing tool. Volumes of interest (VOIs) were manually delineated on selected organs (brain, heart, lungs, liver, kidneys, and bladder). Time‐activity curves (decay corrected) were obtained as cps/cm^3^ in each organ. Curves were transformed into real activity (Bq/cm^3^), and finally injected dose normalization was applied to express the results as percentage of injected dose per cm^3^ of tissue (% ID/cm^3^).

*Ex vivo studies*: After the imaging session, animals were sacrificed, organs of interest were collected and weighed, and the radioactivity was measured in a gamma‐counter (Wallach Wizard, PerkinElmer, Waltham, MA, USA). The uptake was calculated as a percentage of the injected dose per gram of tissue (% ID/g). Then, the weighted organs were immersed in digest solution of HNO_3_/HCl (4 : 1, 5 mL) and heated to boiling until complete dissolution. The solution was subsequently analyzed by ICP‐MS to determine the concentration of Pd in each sample.

**Statistical analysis**: Differences in concentration of radioactivity in each organ and time points were analyzed using 2‐way ANOVA with Tukey's multiple comparison test. Differences were concluded significant for P values <0. 05: P<0.05, *; P<0.01, **, P<0.001, ***; and P<0.0001, ****. Statistical tests were performed in GraphPad Prism 7.03 (GraphPad Software, CA, USA).

## Conflict of interest

The authors declare no conflict of interest.

1

## Supporting information

As a service to our authors and readers, this journal provides supporting information supplied by the authors. Such materials are peer reviewed and may be re‐organized for online delivery, but are not copy‐edited or typeset. Technical support issues arising from supporting information (other than missing files) should be addressed to the authors.

Supporting Information

## Data Availability

The data that support the findings of this study are available from the corresponding author upon reasonable request.

## References

[1a] K. Lu , T. Aung , N. Guo , R. Weichselbaum , W. Lin , Adv. Mater. 2018, 30, 1707634–1707654; 10.1002/adma.201707634 PMC6586248 29971835

[1b] S. F. M. Van Dongen , S. Cantekin , J. A. A. W. Elemans , A. E. Rowan , R. J. M. Nolte , Chem. Soc. Rev. 2014, 43, 99–122; 24071686 10.1039/c3cs60178a

[1c] T. R. Cook , Y.-R. Zheng , P. J. Stang , Chem. Rev. 2013, 113, 734–777; 23121121 10.1021/cr3002824 PMC3764682

[1d] S. V. Dummert , K. Yadava , H. Saini , M. Z. Hussain , K. Jayaramulu , A. Casini , R. A. Fischer , Chem. Soc. Rev. 2022, 51, 5175–5213; 35670434 10.1039/d1cs00550b

[1e] F. d′Orchymont , J. P. Holland , Angew. Chem. Int. Ed. 2022, DOI: 10.1002/anie.202204072.

[2a] H. Amouri , C. Desmarets , J. Moussa , Chem. Rev. 2012, 112, 2015–2041; 22251425 10.1021/cr200345v

[2b] M. Han , D. M. Engelhard , G. H. Clever , Chem. Soc. Rev. 2014, 43, 1848–1860; 24504200 10.1039/c3cs60473j

[2c] M. D. Ward , C. A. Hunter , N. H. Williams , Acc. Chem. Res. 2018, 51, 2073–2082; 30085644 10.1021/acs.accounts.8b00261

[2d] C. J. Brown , F. D. Toste , R. G. Bergman , K. N. Raymond , Chem. Rev. 2015, 115(9), 3012–3035; 25898212 10.1021/cr4001226

[2e] E. Benchimol , B.-N. T. Nguyen , T. K. Ronson , J. R. Nitschke , Chem. Soc. Rev. 2022, 51, 5101–5135; 35661155 10.1039/d0cs00801j PMC9207707

[2f] Y. Fang , J. A. Powell , E. Li , Q. Wang , Z. Perry , A. Kirchon , X. Yang , Z. Xiao , C. Zhu , L. Zhang , F. Huang , H.-C. Zhou , Chem. Soc. Rev. 2019, 48, 4707–4730; 31339148 10.1039/c9cs00091g

[2g] Y. Sun , C. Chen , J. Liu , P. J. Stang , Chem. Soc. Rev. 2020, 49, 3889–3919; 32412574 10.1039/d0cs00038h PMC7846457

[2h] T. Keijer , T. Bouwens , J. Hessels , J. N. H. Reek , Chem. Sci. 2021, 12, 50–70; 10.1039/d0sc03715j PMC8179670 34168739

[2i] J. Zhao , Z. Zhou , G. Li , P. J. Stang , X. Yan , Natl. Sci. Rev. 2021, 8, nwab045; 34691672 10.1093/nsr/nwab045 PMC8288187

[2j] N. Dey , C. J. E. Haynes , ChemPlusChem 2021, 86, 418; 33665986 10.1002/cplu.202100004

[2k] S. Pullen , G. H. Clever , Acc. Chem. Res. 2018, 51, 3052–3064; 30379523 10.1021/acs.accounts.8b00415 PMC6437652

[2l] X. Jing , C. He , L. Zhao , C. Duan , Acc. Chem. Res. 2019, 52(1), 100–109. 30586276 10.1021/acs.accounts.8b00463

[3a] H. Sepehrpour , W. Fu , Y. Sun , P. J. Stang , J. Am. Chem. Soc. 2019, 141, 14005–14020; 31419112 10.1021/jacs.9b06222 PMC6744948

[3b] B. Therrien , G. Suess-Fink , P. Govindaswamy , A. K. Renfrew , P. J. Dyson , Angew. Chem. Int. Ed. 2008, 47, 3773–3776; 10.1002/anie.200800186 18412203

[3c] J. E. M. Lewis , E. L. Gavey , S. A. Cameron , J. D. Crowley , Chem. Sci. 2012, 3, 778–784;

[3d] Y.-R. Zheng , K. Suntharalingam , T. C. Johnstone , S. J. Lippard , Chem. Sci. 2015, 6, 1189–1193; 25621144 10.1039/c4sc01892c PMC4303084

[3e] W.-Q. Xu , Y.-Z. Fan , H.-P. Wang , J. Teng , Y.-H. Li , C.-X. Chen , D. Fenske , J.-J. Jiang , C.-Y. Su , Chem. Eur. J. 2017, 23, 3542–3547; 28094459 10.1002/chem.201606060

[3f] F. Schmitt , J. Freudenreich , N. P. E. Barry , L. Juillerat-Jeanneret , G. Süss-Fink , B. Therrien , J. Am. Chem. Soc. 2012, 134, 754–757; 22185627 10.1021/ja207784t

[3g] G. Yu , B. Zhu , L. Shao , J. Zhou , M. L. Saha , B. Shi , Z. Zhang , T. Hong , S. Li , X. Chen , P. J. Stang , Proc. Natl. Acad. Sci. USA 2019, 116, 6618–6623; 30894484 10.1073/pnas.1902029116 PMC6452736

[3h] Z. Zhou , J. Liu , J. Huang , T. W. Rees , Y. Wang , H. Wang , X. Li , H. Chao , P. J. Stang , Proc. Natl. Acad. Sci. USA 2019, 116, 20296–20302. 31548389 10.1073/pnas.1912549116 PMC6789806

[4a] B. P. Burke , W. Grantham , M. J. Burke , G. S. Nichol , D. Roberts , I. Renard , R. Hargreaves , C. Cawthorne , S. J. Archibald , P. J. Lusby , J. Am. Chem. Soc. 2018, 140, 16877–16881; 30485075 10.1021/jacs.8b09582

[4b] H. Zhu , Q. Li , B. Shi , F. Ge , Y. Liu , Z. Mao , H. Zhu , S. Wang , G. Yu , F. Huang , P. J. Stang , Angew. Chem. Int. Ed. 2020, 59, 20208–20214; 10.1002/anie.202009442 32710650

[5a] J. Xu , J. Wang , J. Ye , J. Jiao , Z. Liu , C. Zhao , B. Li , Y. Fu , Adv. Sci. 2021, 8, 2101101; 10.1002/advs.202101101 PMC8373122 34145984

[5b] G. Yua , T. R. Cookb , Y. Lic , X. Yand , D. Wuc , L. Shaoa , J. Shenc , G. Tangc , F. Huanga , X. Chene , P. J. Stang , Proc. Nat. Acad. Sci. 2016, 113, 13720–13725; 27856738 10.1073/pnas.1616836113 PMC5137737

[5c] G. Yu , S. Yu , M. L. Saha , J. Zhou , T. R. Cook , B. C. Yung , J. Chen , Z. Mao , F. Zhang , Z. Zhou , Y. Liu , L. Shao , S. Wang , C. Gao , F. Huang , P. J. Stang , X. Chen , Nat. Commun. 2018, 9, 1–18. 30337535 10.1038/s41467-018-06574-7 PMC6194061

[6a] A. Schmidt , V. Molano , M. Hollering , A. Pöthig , A. Casini , F. E. Kühn , Chem. A Eur. J. 2016, 22, 2253–2256; 10.1002/chem.201504930 26756963

[6b] B. Woods , M. N. Wenzel , T. Williams , S. R. Thomas , R. L. Jenkins , A. Casini , Front. Chem. 2019, 7, 68. 30834242 10.3389/fchem.2019.00068 PMC6387950

[7] A. Schmidt , M. Hollering , M. Drees , A. Casini , F. E. Kühn , Dalton Trans. 2016, 45, 8556. 27126799 10.1039/c6dt00654j

[8] A. Schmidt , M. Hollering , J. Han , A. Casini , F. E. Kühn , Dalton Trans. 2016, 45, 12297. 27436541 10.1039/c6dt02708c

[9a] B. Woods , D. Döllerer , B. Aikman , M. N. Wenzel , E. Sayers , F. E. Kühn , A. Jones , A. Casini , J. Inorg. Biochem. 2019, 199, 110781; 31357067 10.1016/j.jinorgbio.2019.110781

[9b] B. Aikman , R. Bonsignore , B. Woods , D. Doellerer , R. Scotti , C. Schmidt , A. A. Heidecker , A. Pöthig , E. J. Sayers , A. T. Jones , A. Casini , Dalton Trans. 2022, DOI: 10.1039/D2DT00337F. 35470841

[10] J. Han , A. Schmidt , T. Zhang , H. Permentier , G. M. M. Groothuis , R. Bischoff , F. E. Kühn , P. Horvatovich , A. Casini , Chem. Commun. 2017, 53, 1405–1408. 10.1039/c6cc08937b 28079225

[11] J. Han , A. F. B. Räder , F. Reichart , B. Aikman , M. N. Wenzel , B. Woods , M. Weinmüller , B. S. Ludwig , S. Stürup , G. M. M. Groothuis , H. P. Permentier , R. Bischoff , H. Kessler , P. Horvatovich , A. Casini , Bioconjugate Chem. 2018, 29, 3856–3865. 10.1021/acs.bioconjchem.8b00682 30380298

[12] B. Woods , R. D. M. Silva , C. Schmidt , D. Wragg , M. Cavaco , V. Neves , V. F. C. Ferreira , L. Gano , T. S. Morais , F. Mendes , J. D. G. Correia , A. Casini , Bioconjugate Chem. 2021, 32, 1399–1408. 10.1021/acs.bioconjchem.0c00659 33440122

[13a] A. Casini , B. Woods , M. Wenzel , Inorg. Chem. 2017, 56, 14715–14729; 29172467 10.1021/acs.inorgchem.7b02599

[13b] A. Pöthig , A. Casini , Theranostics 2019, 9, 3150–3169. 31244947 10.7150/thno.31828 PMC6567972

[14a] Z. Liu , M. Pourghiasian , M. A. Radtke , J. Lau , J. Pan , G. M. Dias , D. Yapp , K−S. Lin , F. Bénard , D. M. Perrin , Angew. Chem. Int. Ed. 2014, 53, 11876–11880; 10.1002/anie.201406258 25196467

[14b] D. M. Perrin , Curr. Opin. Chem. Biol. 2018, 45, 86–94; 29684871 10.1016/j.cbpa.2018.03.001

[14c] A. Roxin , C. Zhang , S. Huh , M. Lepage , Z. Zhang , K−S. Lin , F. Bénard , D. M. Perrin , Bioconjugate Chem. 2019, 30, 1210–1219. 10.1021/acs.bioconjchem.9b00146 30896929

[15] M. E. Bakkari , J.-M. Vincent , Org. Lett. 2004, 6, 2765–2767. 15281764 10.1021/ol048994b

[16] H. Pohlit , M. Worm , J. Langhanki , E. Berger-Nicoletti , T. Opatz , H. Frey , Macromolecules 2017, 50, 9196–9206.

[17] D. H. Leung , R. G. Bergman , K. N. Raymond , J. Am. Chem. Soc. 2008, 130, 2798–2805. 18257565 10.1021/ja075975z

[18] D. Preston , S. M. McNeill , J. E. M. Lewis , G. I. Giles , J. D. Crowley , Dalton Trans. 2016, 45, 8050–8060. 27074828 10.1039/c6dt00133e

[19a] E. Puig , C. Desmarets , G. Gontard , M. N. Rager , A. L. Cooksy , H. Amouri , Inorg. Chem. 2019, 58, 3189–3195; 30762339 10.1021/acs.inorgchem.8b03272

[19b] F. Kaiser , A. Schmidt , W. Heydenreuter , P. J. Altmann , A. Casini , S. A. Sieber , F. E. Kühn , Eur. J. Inorg. Chem. 2016, 33, 5189–5196.

[20] J. B. Williamson , E. R. Smith , J. R. Scheerer , Synlett. 2017, 28, 1170–1172. 29398786 10.1055/s-0036-1588729 PMC5791744

