# Supplementary material for: PET Imaging of Self‐Assembled 18F‐Labelled Pd2L4 Metallacages for Anticancer Drug Delivery
Source: Chemistry. 2022 Nov 23;29(3):e202202604. doi: 10.1002/chem.202202604 (PMC10168593; doi:10.1002/chem.202202604)
Supplement: Supplementary file 1 — Supporting Information [file CHEM-29-0-s001.pdf]

Chemistry–A European Journal

Supporting Information

PET Imaging of Self-Assembled ^{18}F-Labelled Pd_2L_4 Metallacages for Anticancer Drug Delivery

Raúl Cosialls, Cristina Simó, Salvador Borrós, Vanessa Gómez-Vallejo, Claudia Schmidt, Jordi Llop,* Ana B. Cuenca,* and Angela Casini*

Table of Contents

1. Synthesis and characterization of $AMBF_3$-modified metallacages 3

1.1 Synthetic scheme for the preparation of precursors 1 and 2’ 3

1.2 NMR of azide precursor 2 4

1.3 NMR of ligand L1 5

1.4 NMR of ligand L2 7

1.5 NMR of metallacage C1 9

1.6 NMR of metallacage C2 11

1.7 ^{1}H DOSY NMR 13

1.8 High-resolution ESI mass spectrometry 13

2. Encapsulation studies 17

2.1 Experimental protocols 17

2.2 ^{1}H NMR spectroscopy 18

2.3 ^{195}Pt NMR spectroscopy 19

2.4 High resolution ESI mass spectrometry of cisplatin loaded C1 19

2.5 High resolution ESI mass spectrometry of cisplatin loaded C2 21

3. Stability experiments 24

3.1 ^{1}H NMR spectroscopy 24

3.2 High-resolution ESI mass spectrometry 25

4. Radiolabelling and biodistribution studies 28

4.1 Radiochemistry 28

4.2 *In vivo* and *Ex vivo* studies 29

1. Synthesis and characterization of AMBF3-modified metallacages

1.1 Synthetic scheme for the preparation of precursors **1** and **2'**

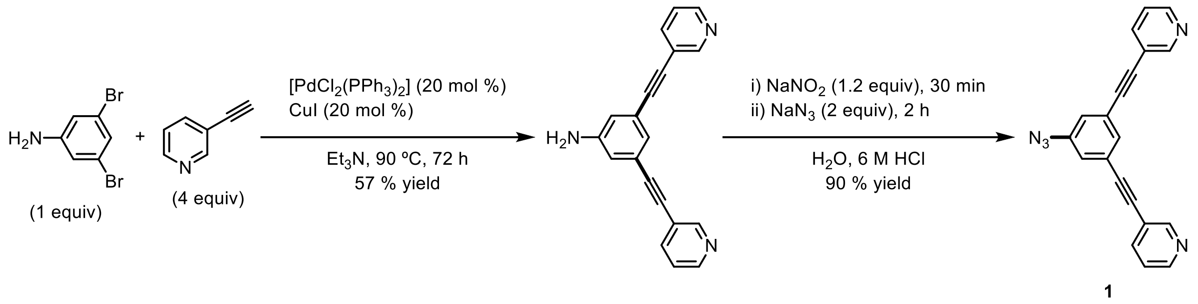

Figure S1a. Synthesis of azide precursor **1**.

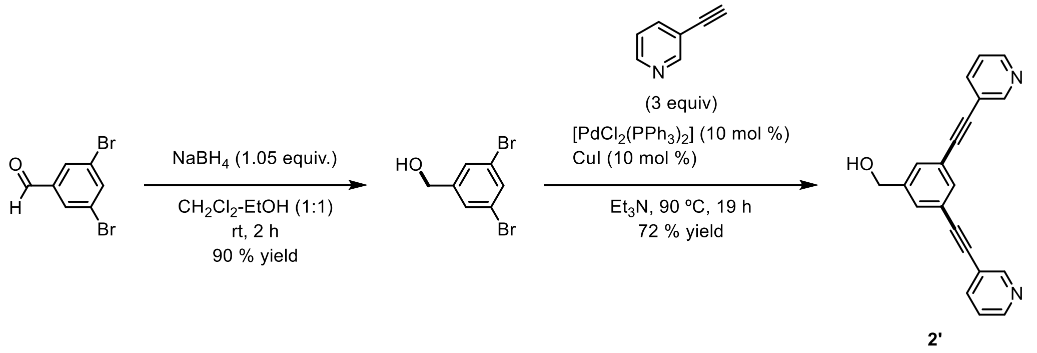

Figure S1b. Synthesis of alcohol precursor **2'**.

1.2 NMR of azide precursor **2**

^{1}H NMR (DMSO-d_6, 400 MHz)

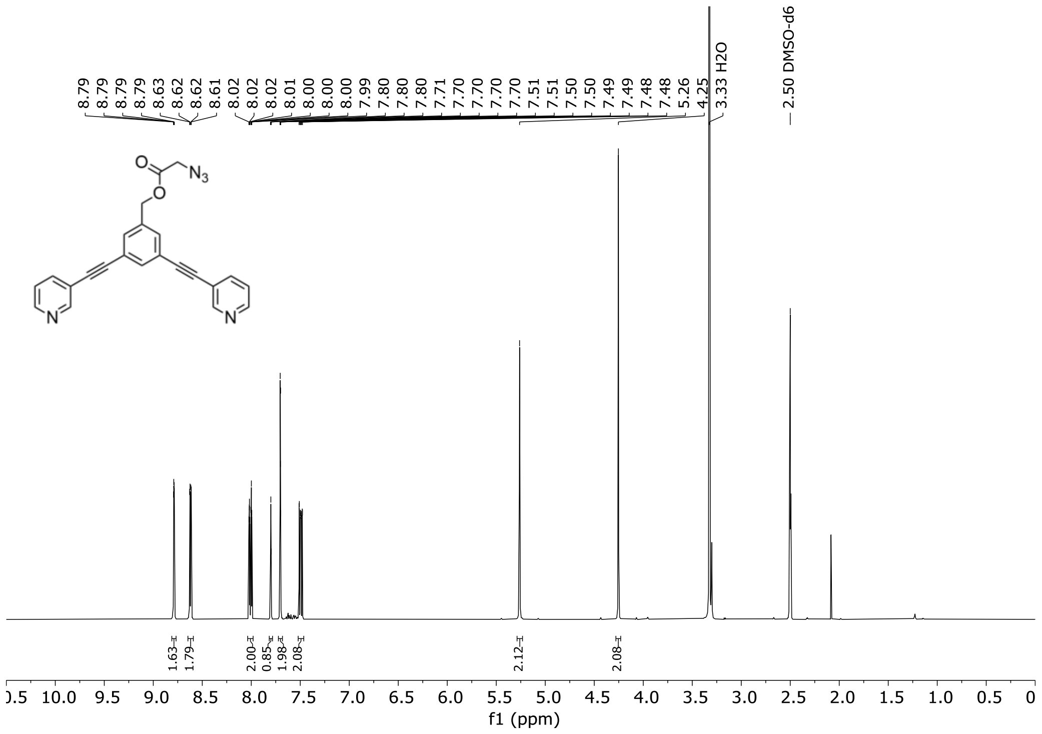

^{13}C NMR (DMSO-d_6, 100 MHz)

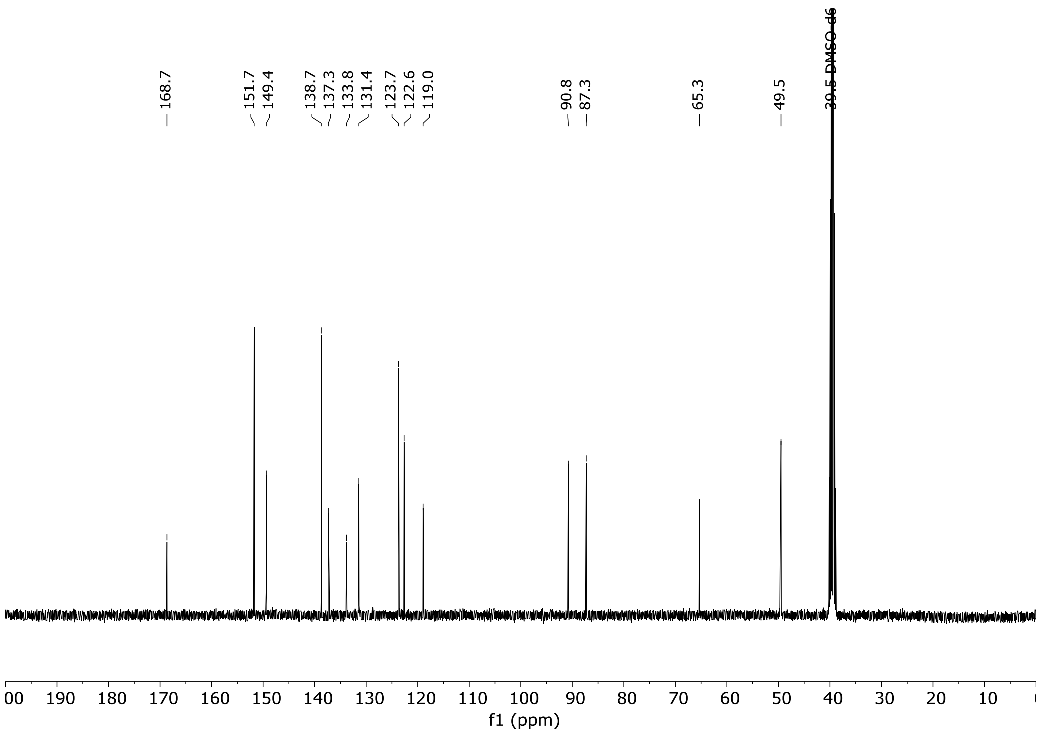

Figure S2. Characterization of azide precursor **2** by ^{1}H and ^{13}C NMR in DMSO-d_6.

1.3 NMR of ligand **L1**

^{1}H NMR (DMSO-d_6, 400 MHz)

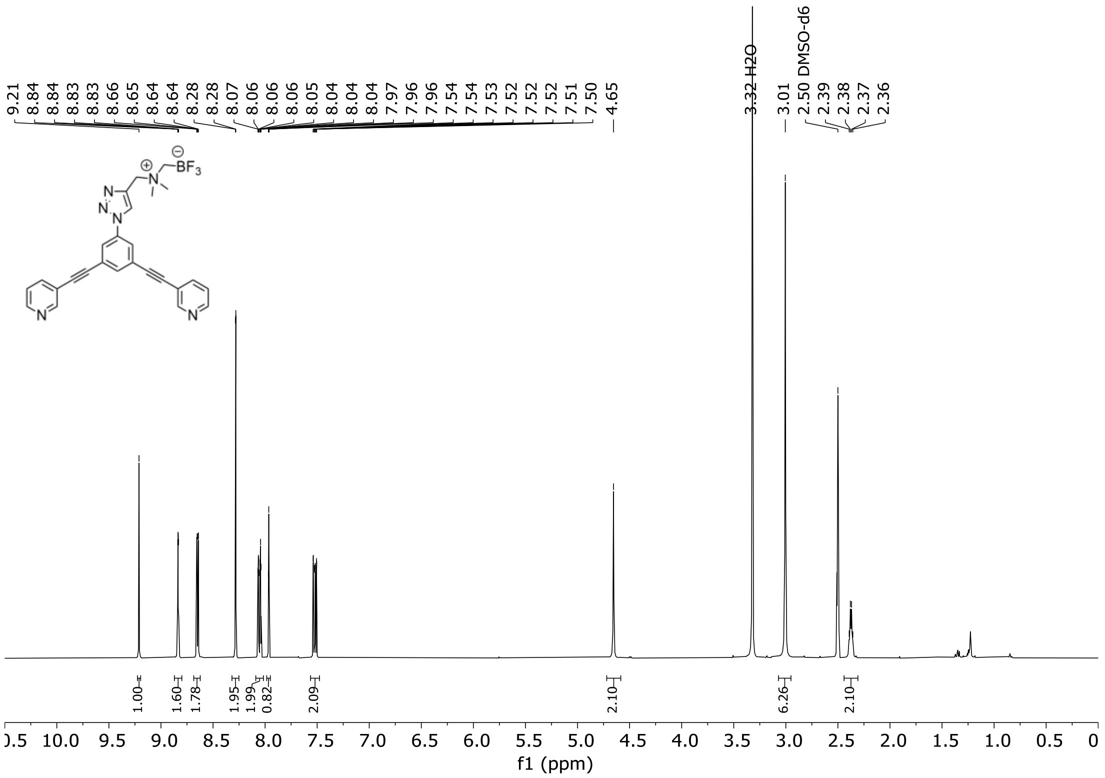

^{13}C NMR (DMSO-d_6, 100 MHz)

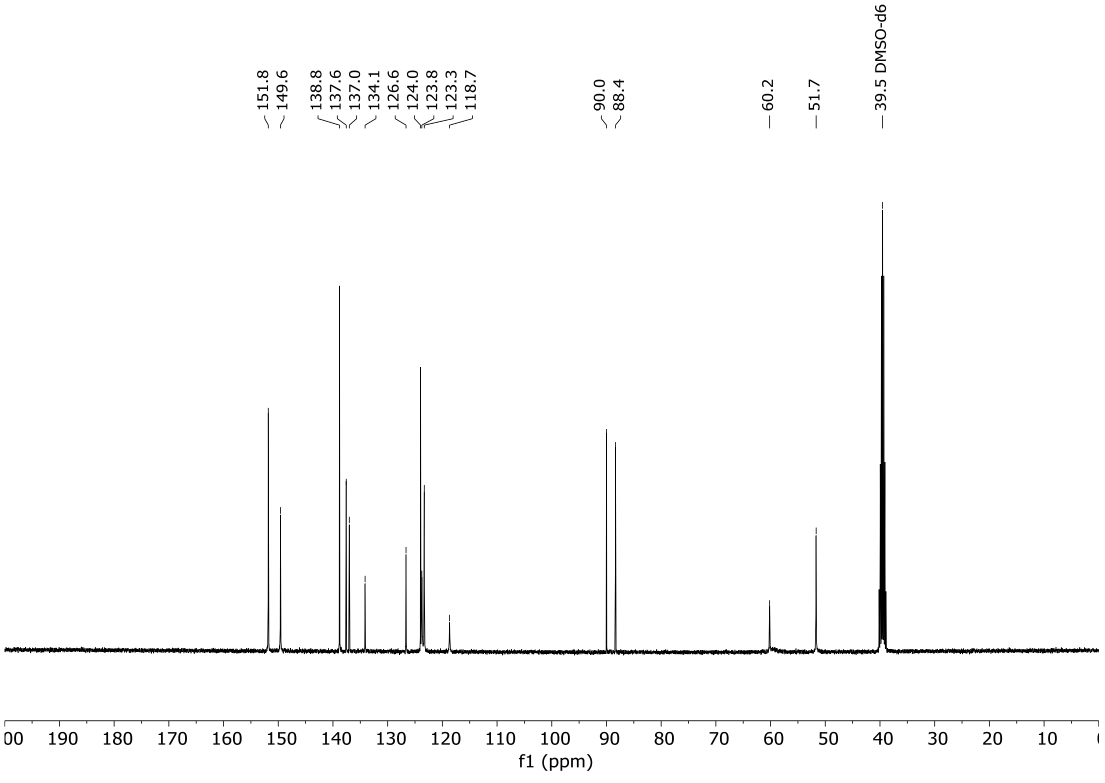

^{19}F NMR (DMSO-d_6, 376 MHz)

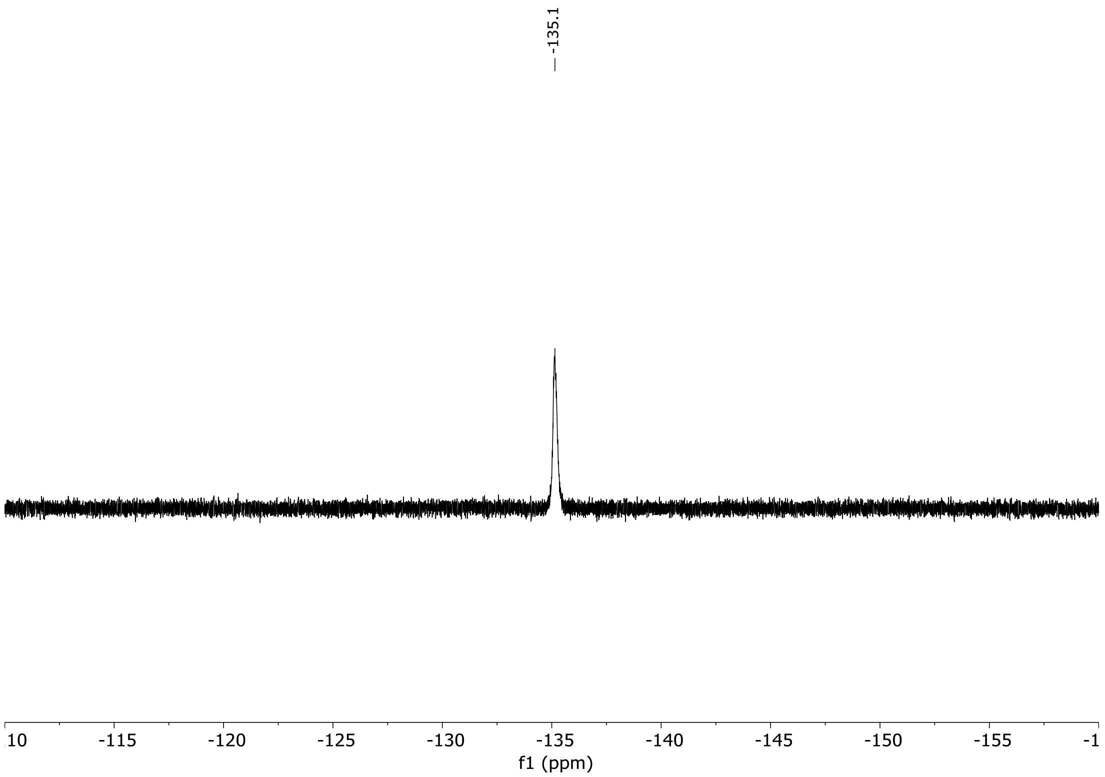

^{11}B NMR (DMSO-d_6, 128 MHz)

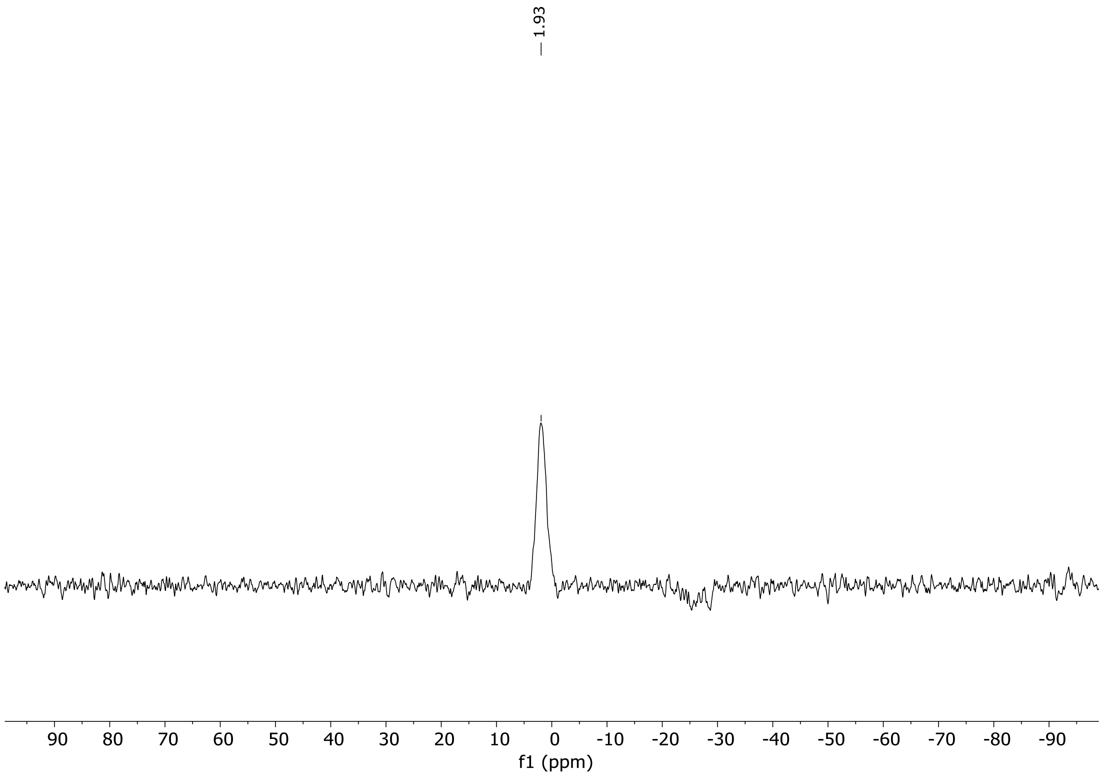

Figure S3. Characterization of ligand **L1** by ^{1}H, ^{13}C, ^{19}F and ^{11}B NMR in DMSO-d_6.

1.4 NMR of ligand L2

^{1}H NMR (DMSO-d_6, 400 MHz)

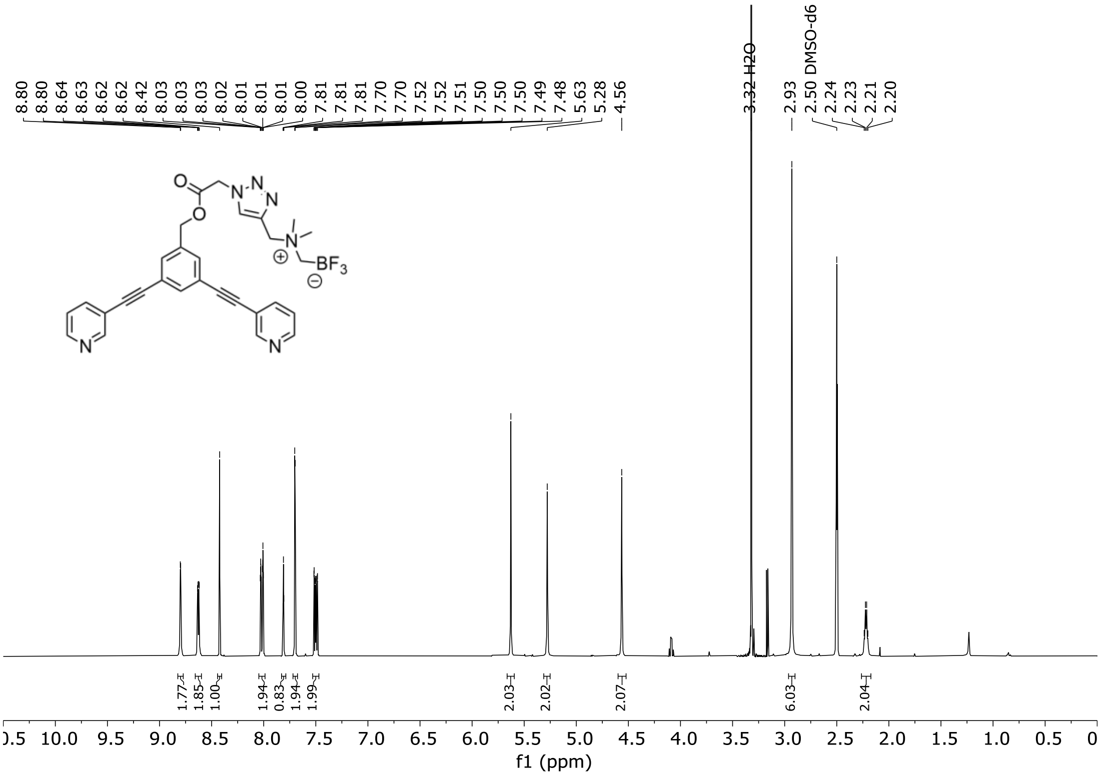

^{13}C NMR (DMSO-d_6, 100 MHz)

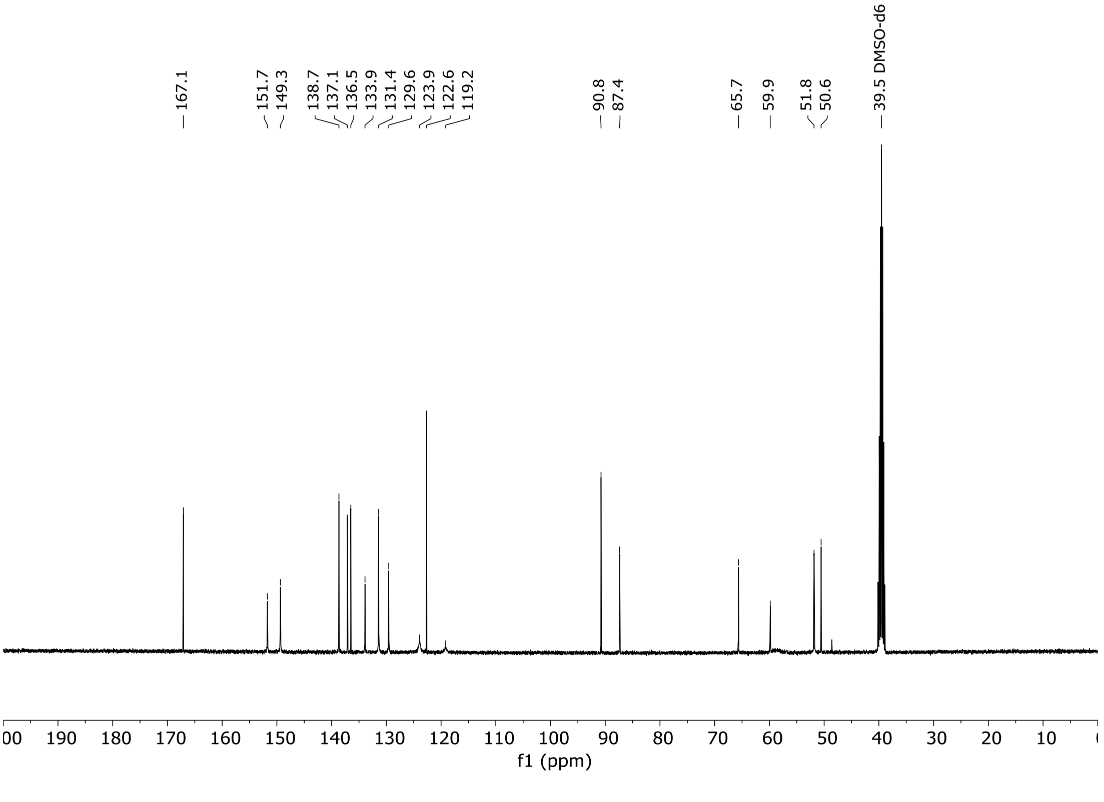

^{19}F NMR (DMSO-d_6, 376 MHz)

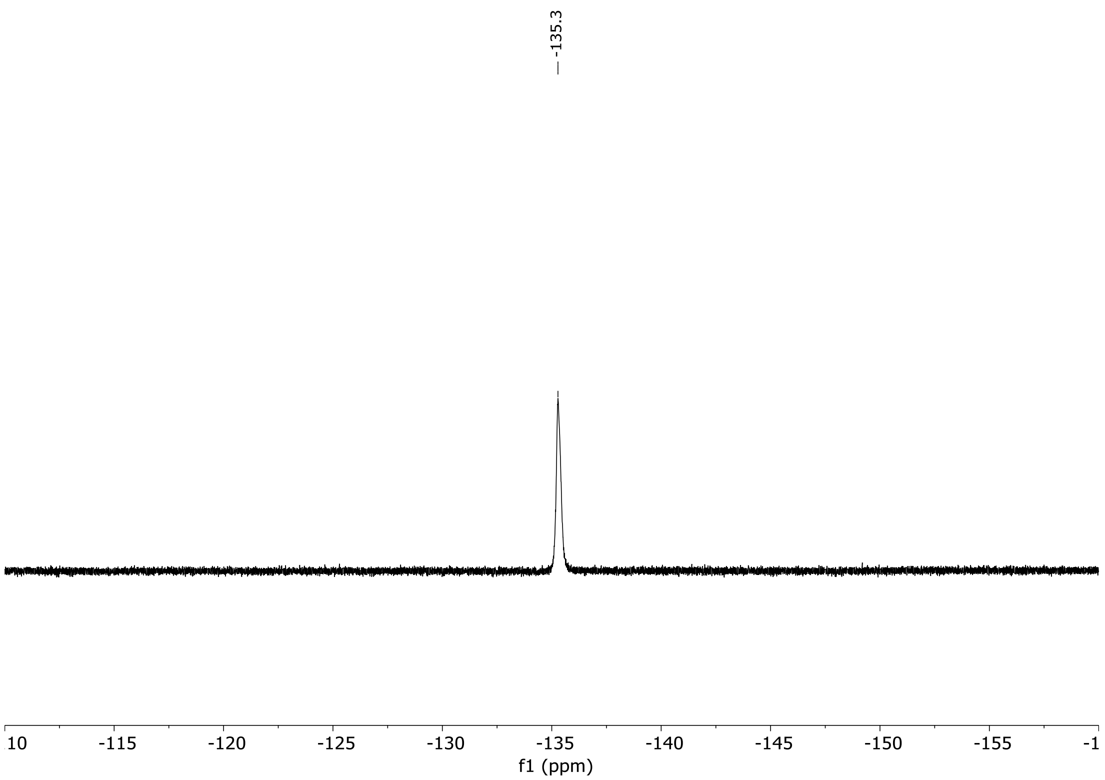

^{11}B NMR (DMSO-d_6, 128 MHz)

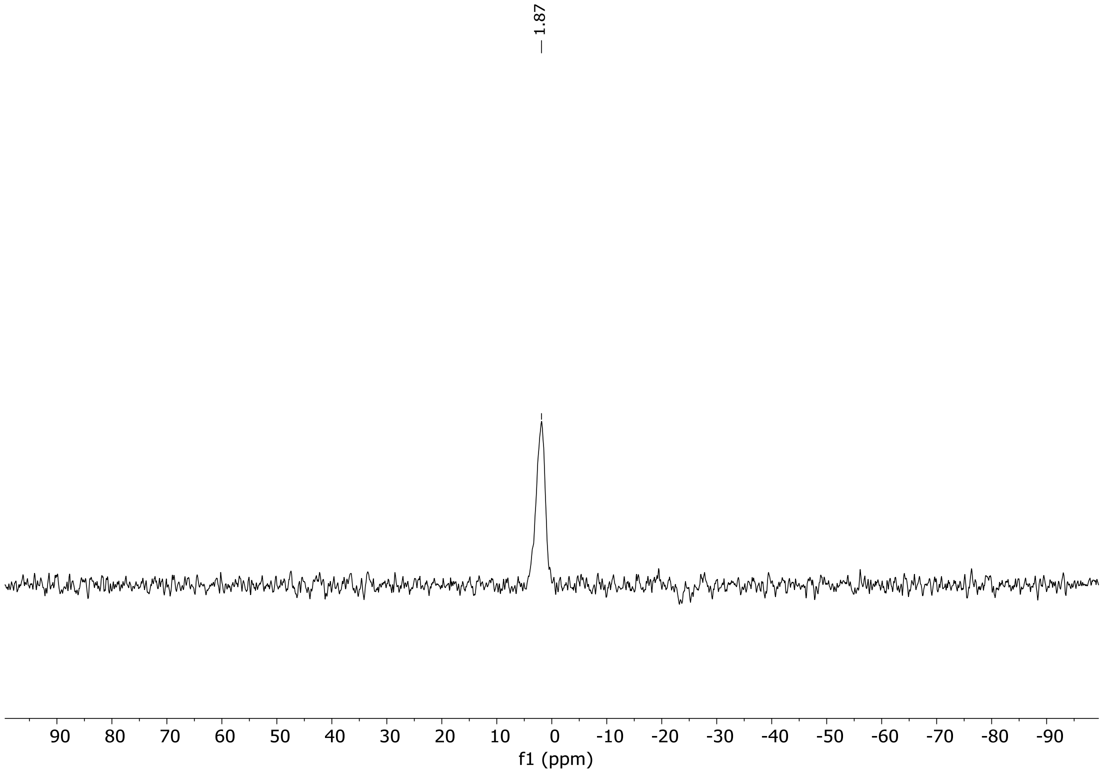

Figure S4. Characterization of ligand **L2** by ^{1}H, ^{13}C, ^{19}F and ^{11}B NMR in DMSO-d_6.

1.5 NMR of metallacage **C1**

^{1}H NMR (DMSO-d_6, 400 MHz)

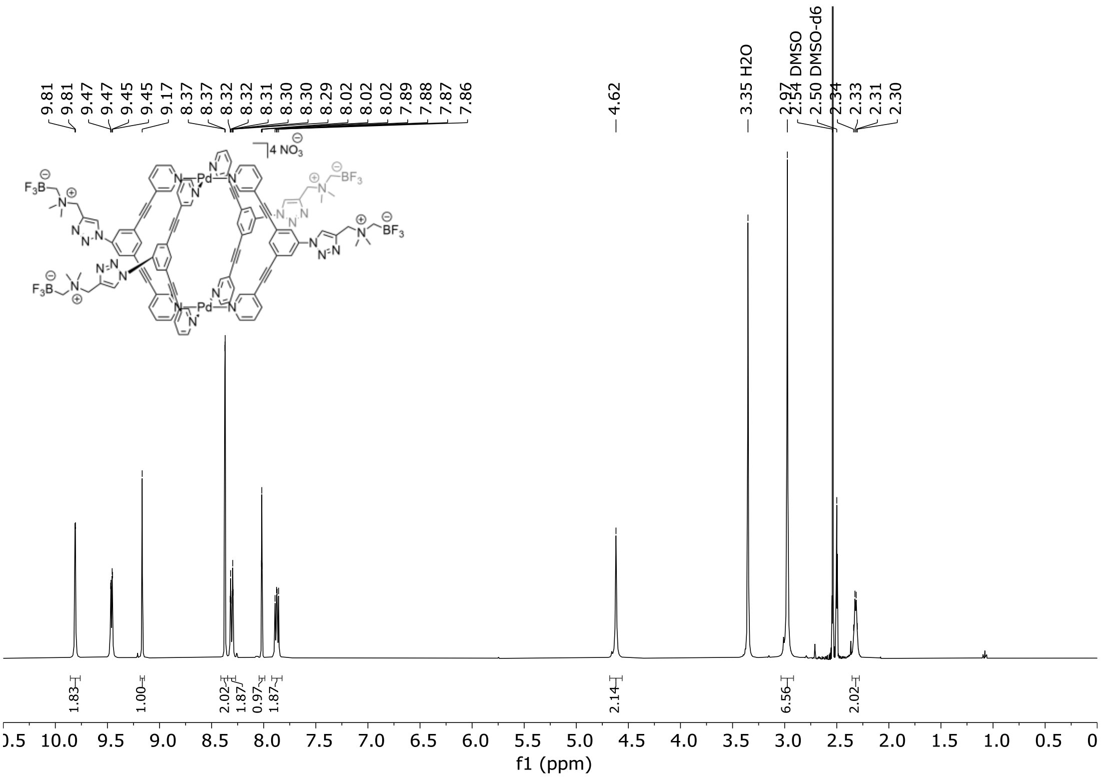

^{13}C NMR (DMSO-d_6, 100 MHz)

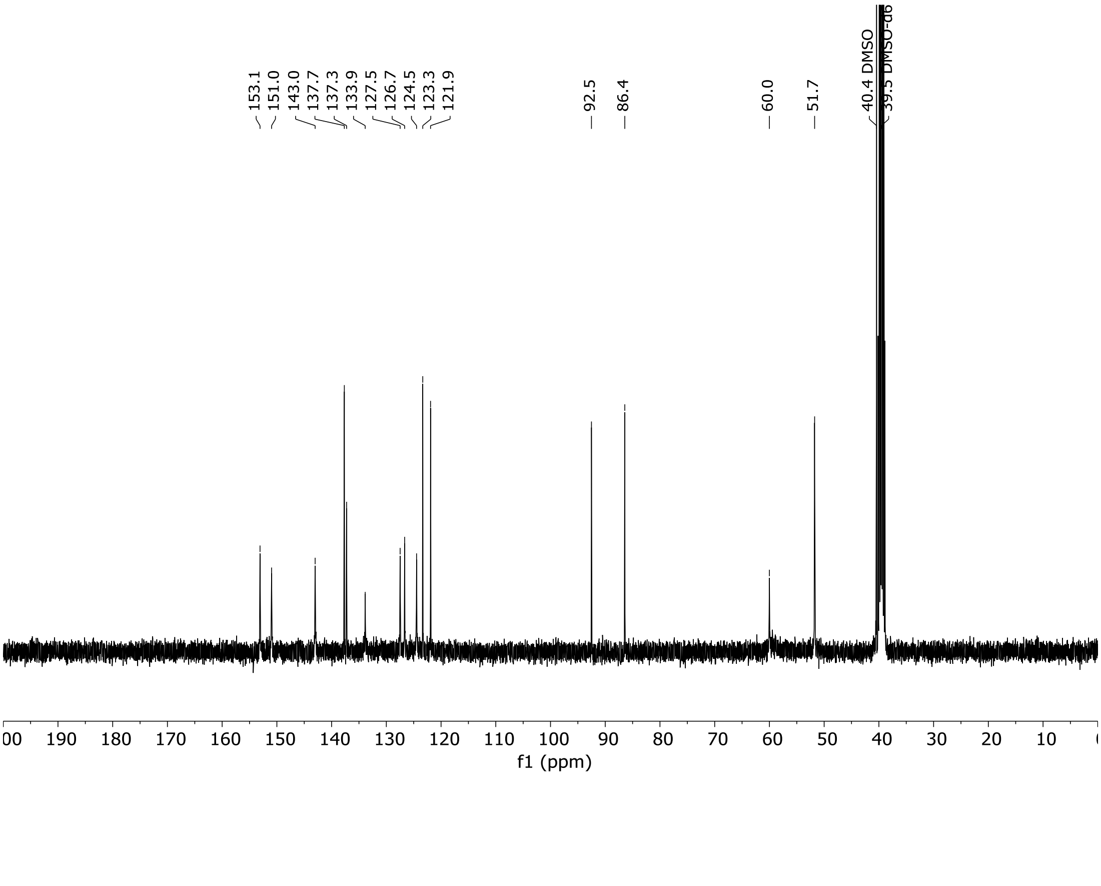

^{19}F NMR (DMSO-d_6, 376 MHz)

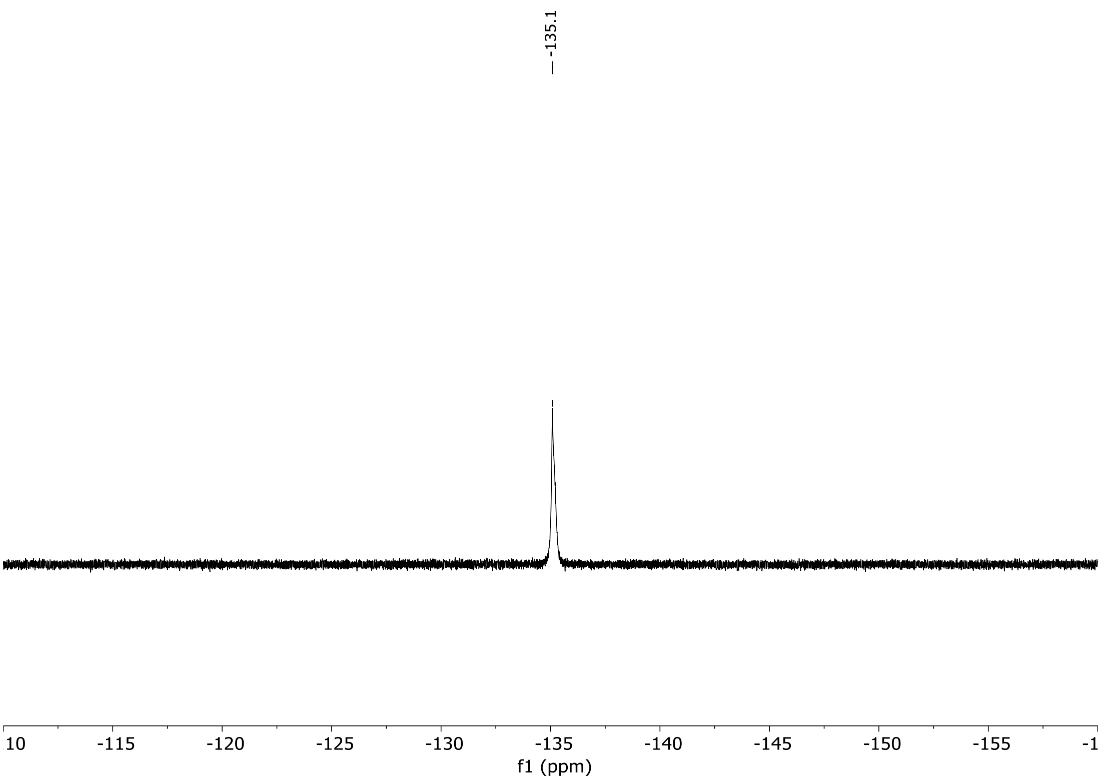

^{11}B NMR (DMSO-d_6, 128 MHz)

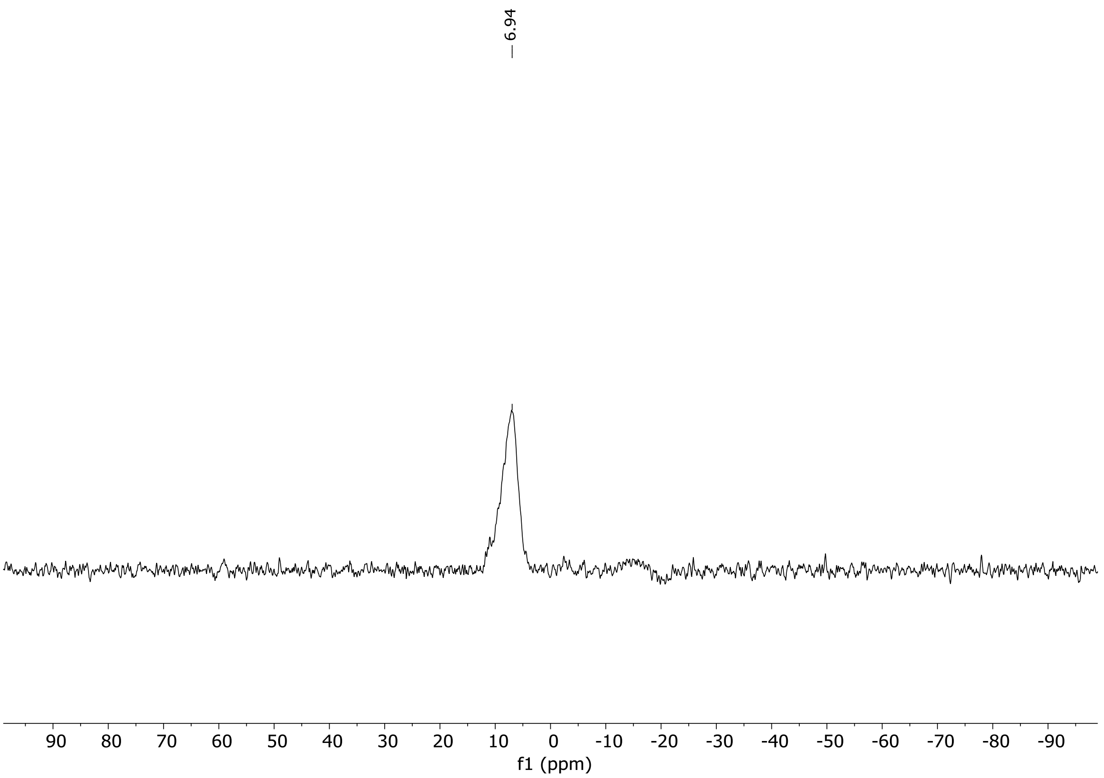

Figure S5. Characterization of metallacage **C1** by ^{1}H, ^{13}C, ^{19}F and ^{11}B NMR in DMSO-d_6.

1.6 NMR of metallacage **C2**

^1H NMR (DMSO-d_6, 400 MHz)

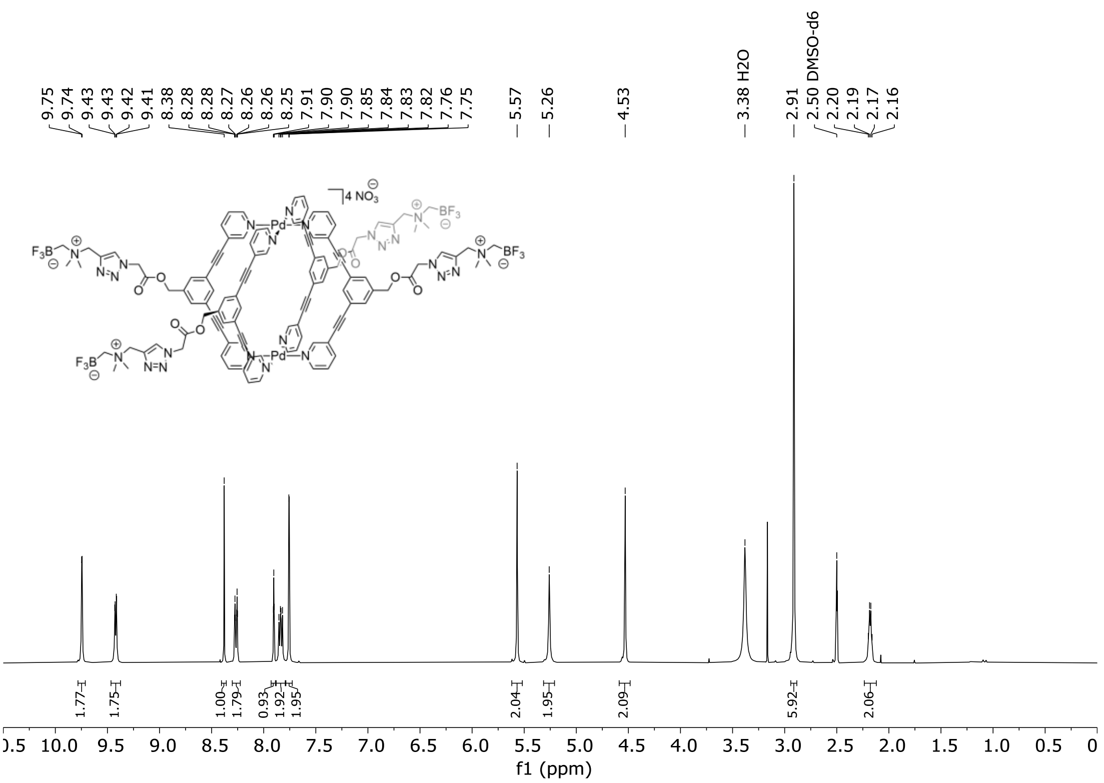

^{13}C NMR (DMSO-d_6, 100 MHz)

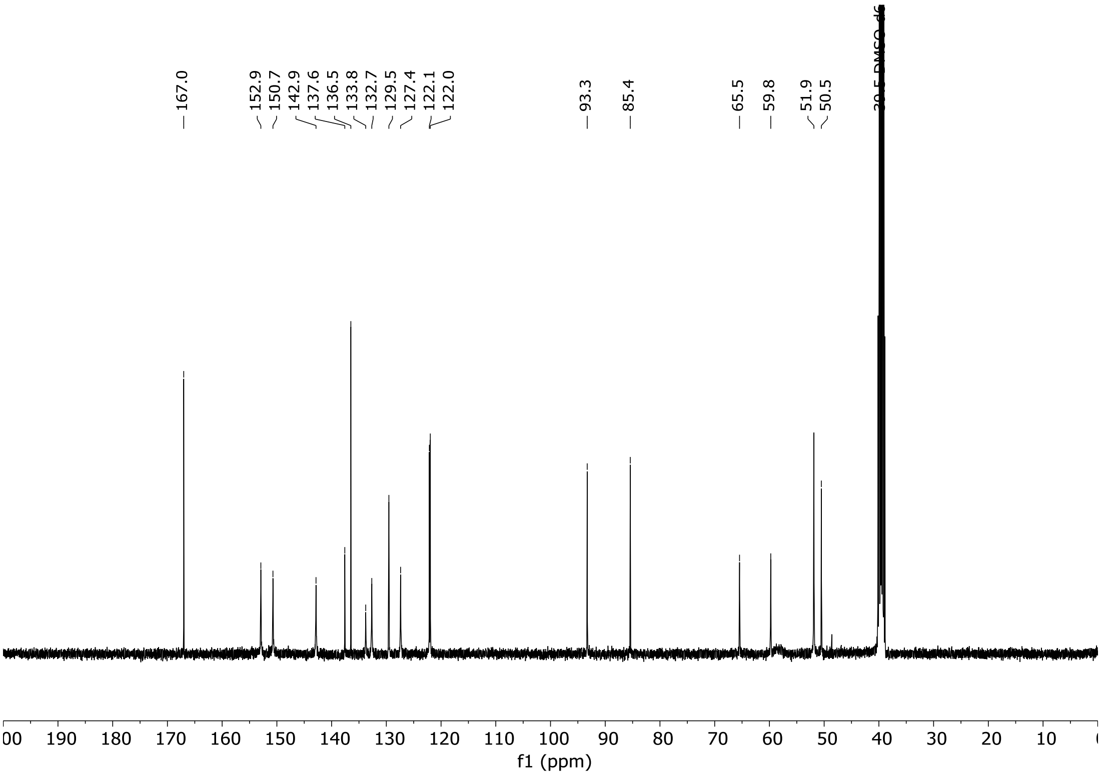

^{19}F NMR (DMSO-d_6, 376 MHz)

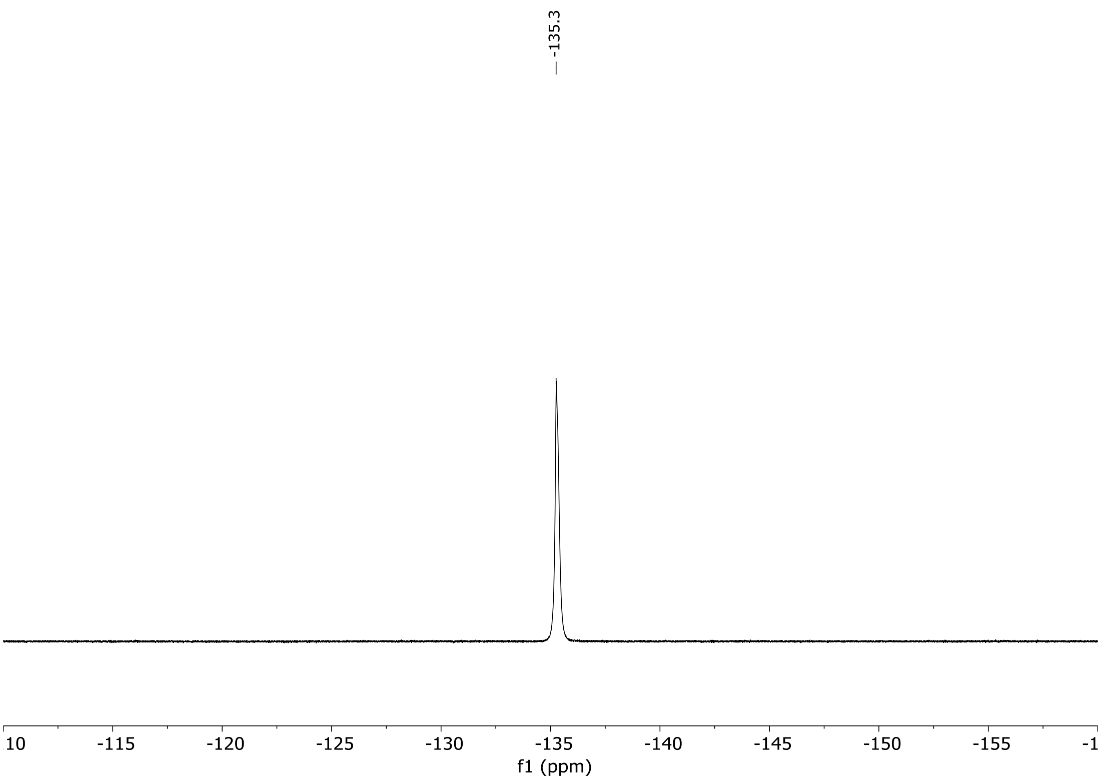

^{11}B NMR (DMSO-d_6, 128 MHz)

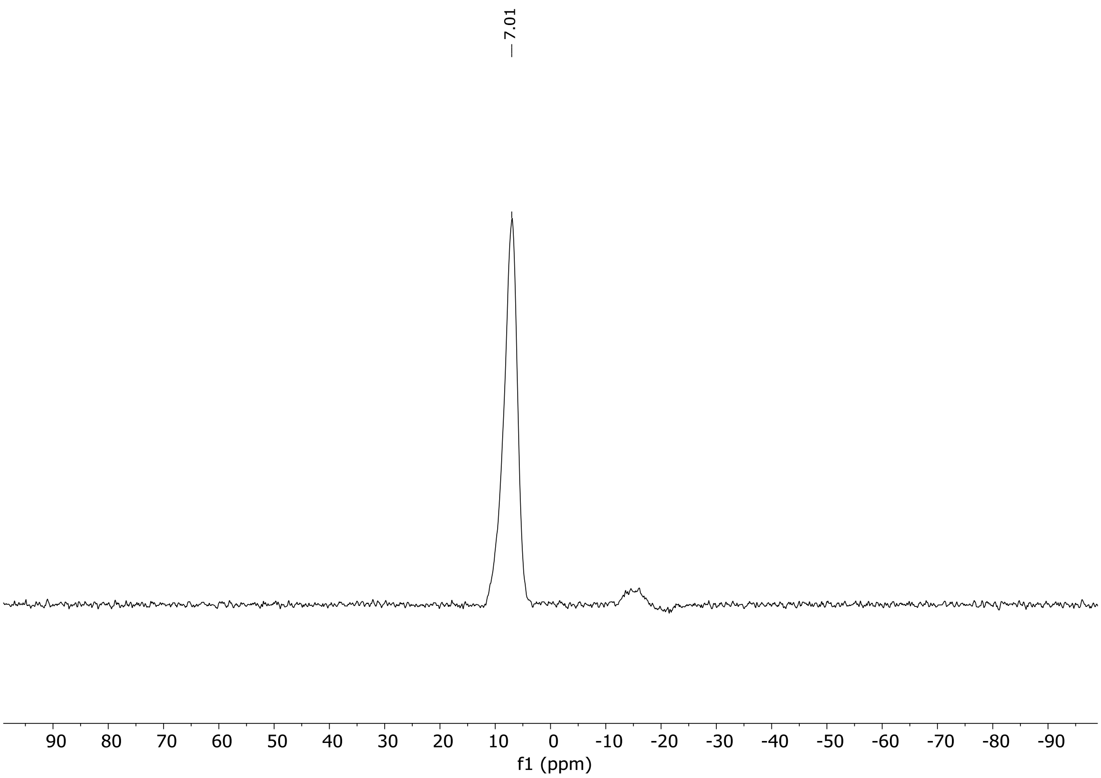

Figure S6. Characterization of metallacage **C2** by ^{1}H, ^{13}C, ^{19}F and ^{11}B NMR in DMSO-d_6.

1.7 ^{1}H DOSY NMR

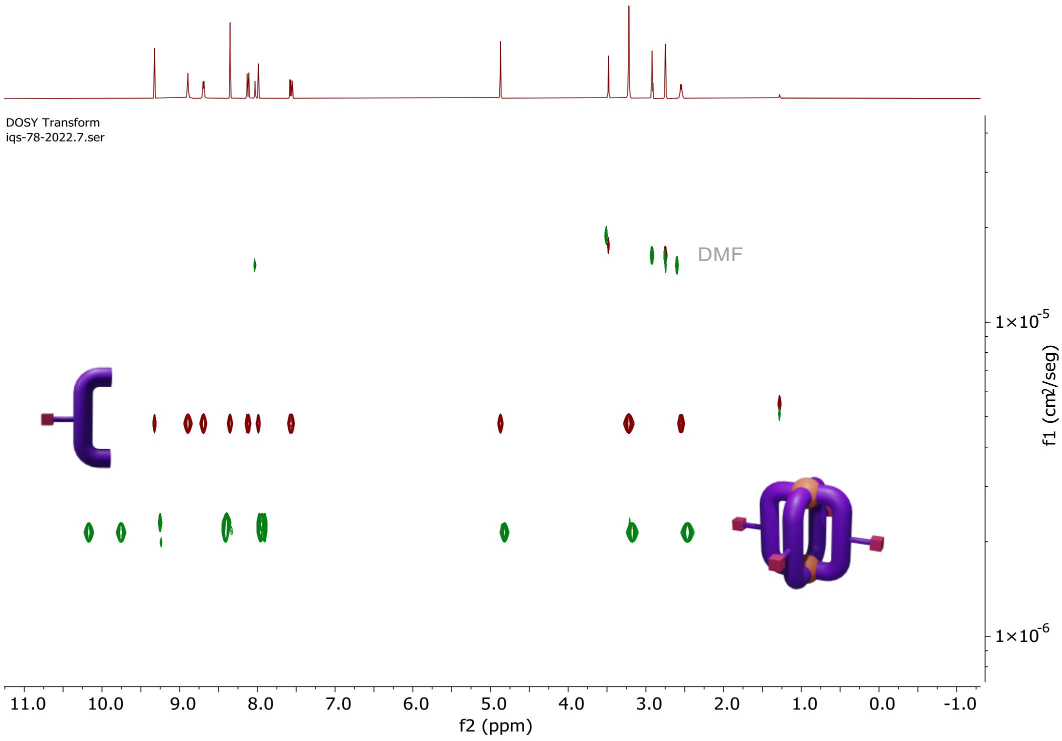

Figure S7. Superposition of ^{1}H NMR DOSY of **L1** (dark red, 4.75x10^{-6} cm^{2}/seg) and **C1** (green, 2.14x10^{-6} cm^{2}/seg) in DMF-d_7.

1.8 High-resolution ESI mass spectrometry

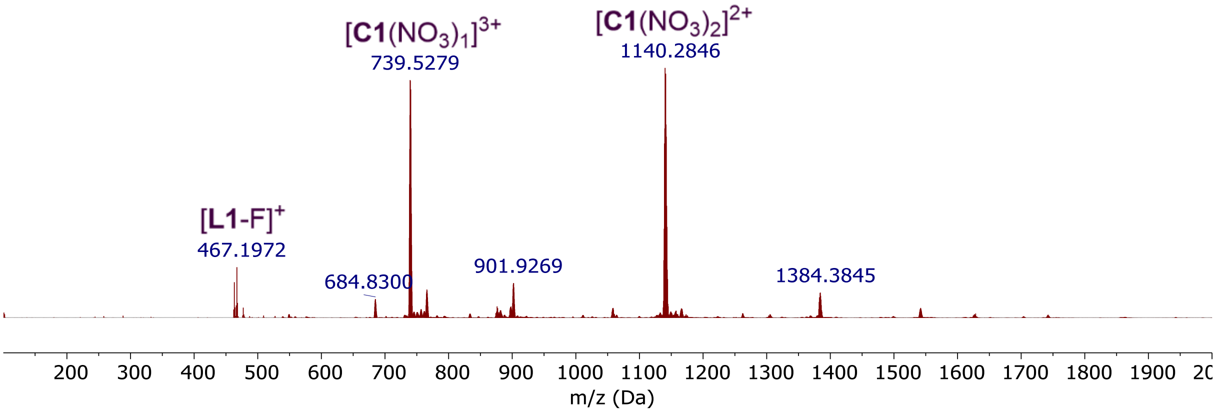

Figure S8a. High-resolution mass spectrum of **C1** in DMSO (direct inject., + mode, 80 dp).

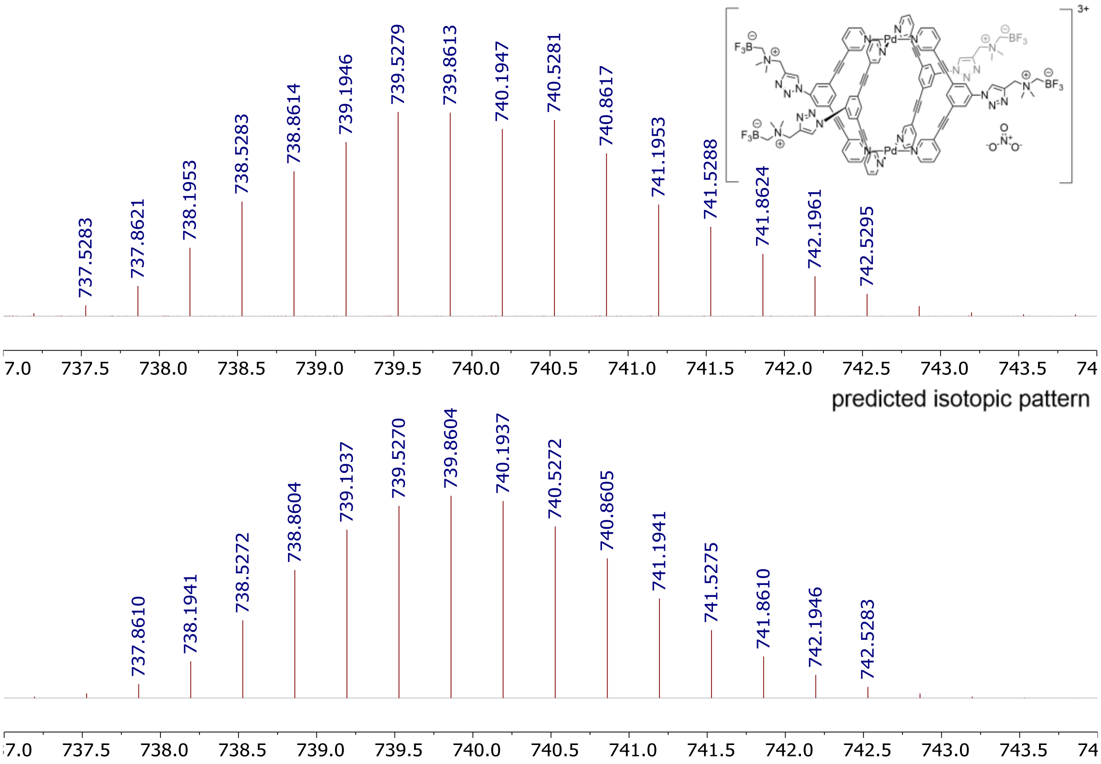

Figure S8b. Experimental (top) and predicted (bottom) isotope patterns for [**C1**$(NO_3)_1$]$^{3+}$ observed by high-resolution mass spectrum of **C1** in DMSO (direct inject., + mode, 80 dp).

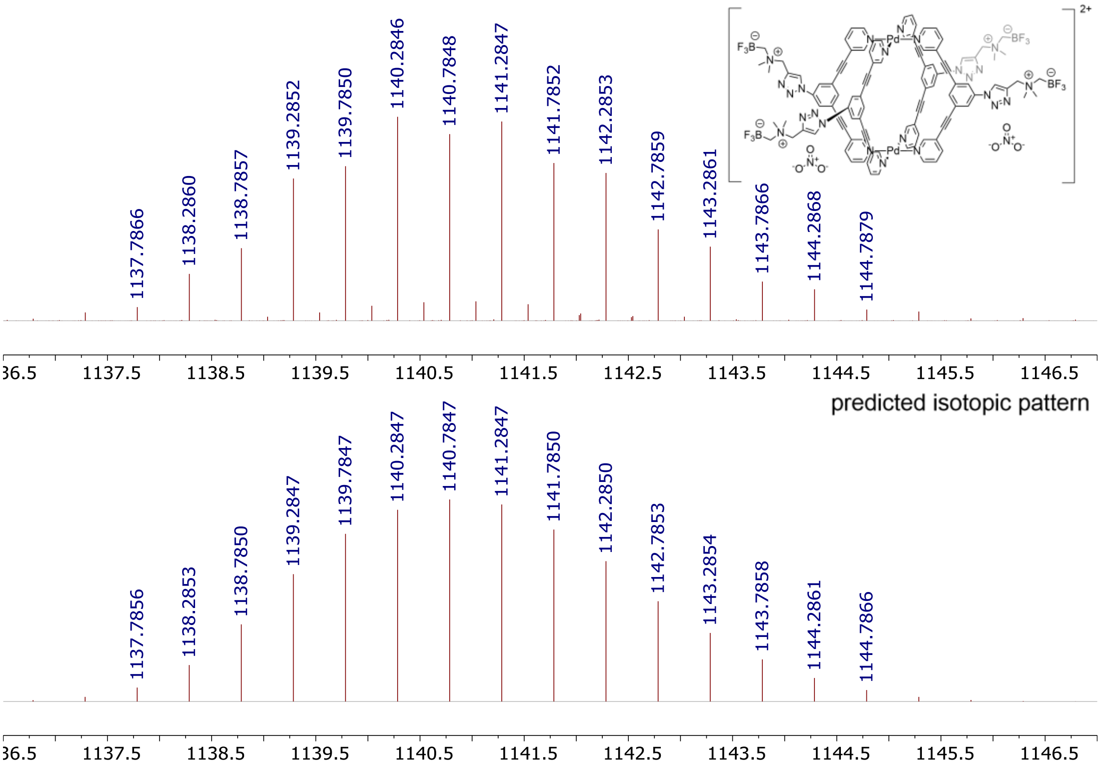

Figure S8c. Experimental (top) and predicted (bottom) isotope patterns for [**C1**$(NO_3)_2$]$^{2+}$ observed by high-resolution mass spectrum of **C1** in DMSO (direct inject., + mode, 80 dp).

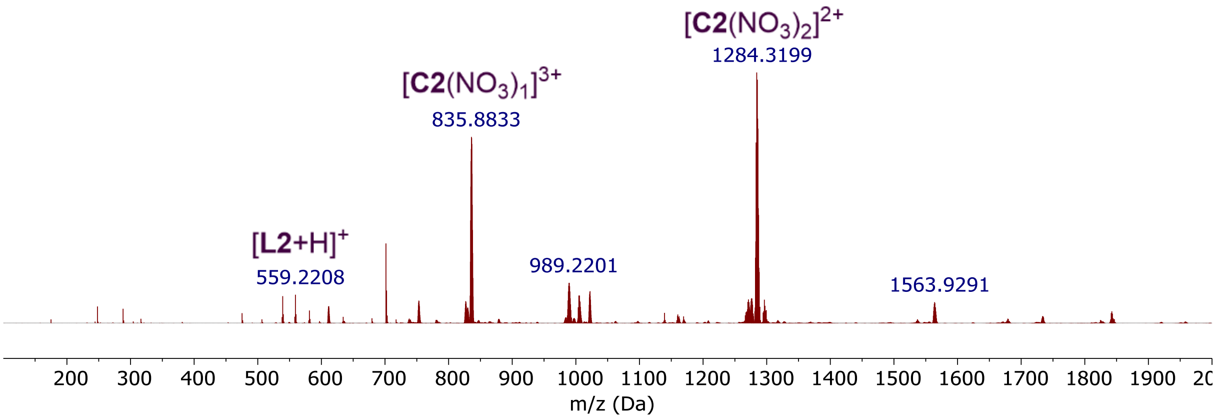

Figure S9a. High-resolution mass spectrum of **C2** in DMF (direct inject., + mode, 80 dp).

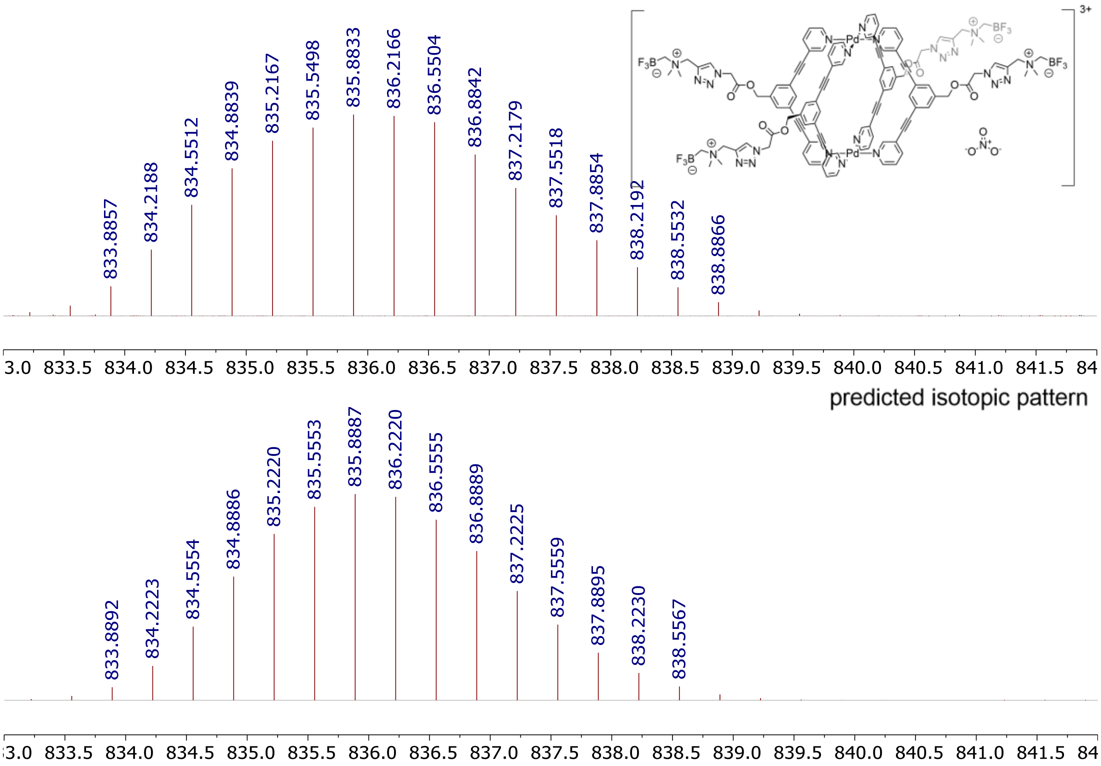

Figure S9b. Experimental (top) and predicted (bottom) isotope patterns for [**C2**$(NO_3)_1]^{3+}$ observed by high-resolution mass spectrum of **C2** in DMF (direct inject., + mode, 80 dp).

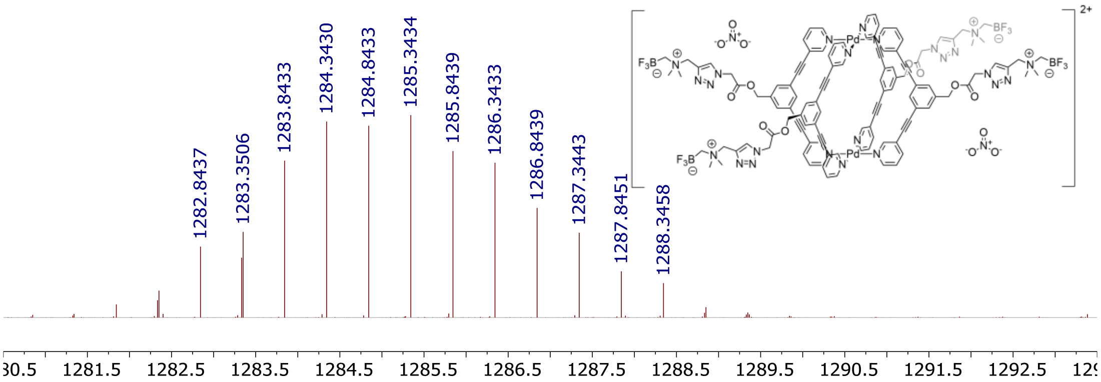

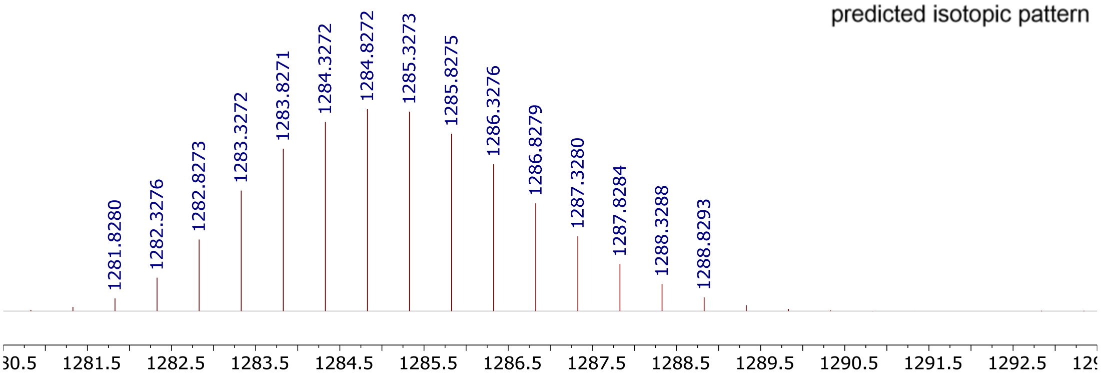

Figure S9c. Experimental (top) and predicted (bottom) isotope patterns for [**C2**$(NO_3)_2$]$^{2+}$ observed by high-resolution mass spectrum of **C2** in DMF (direct inject., + mode, 130 dp).

2. Encapsulation studies

2.1 Experimental protocols

Encapsulation process studied by ^1H NMR

In a 1.5 mL Eppendorf with 8.0 mg **C1** (or 9.0 mg **C2**) (3.3 µmol, 1 equiv.), 0.5 mL of DMF-d_7 were added and the mixture was sonicated for 10 min. The solution was transferred to an NMR tube and a ^1H NMR spectra was recorded (64 scans, 5 s relaxation time). Then, the solution was transferred to an Eppendorf with cisplatin (1.0 mg, 3.3 µmol, 1 equiv.), and was subsequently sonicated for another 10 min. Next, a ^1H NMR spectra was recorded again. This procedure was repeated with the second and with a third equivalent of cisplatin.

Encapsulation process studied by ^{195}Pt NMR

The ^{195}Pt NMR of a solution of cisplatin (5 mg, 16.6 µmol, 2 equiv.) in DMF-d_7 (0.6 mL) was initially recorded. Next, this solution was transferred to an Eppendorf containing 1 equiv. of **C1** (20 mg, 8.3 µmol), the mixture was homogenized and a ^{195}Pt NMR was recorded again.

Encapsulation process studied by high-resolution ESI mass spectrometry

The final solution with 3 equiv. of cisplatin obtained after the encapsulation study by ^1H NMR was diluted with DMF, filtered through a 0.22 µm nylon filter and analyzed. The samples in injection concentration were freshly prepared by adding 25 µL of 10 mM solution of **C1** or **C2** in DMSO to 0.5 mL of 1 mM solution of cisplatin in milliQ water.

^1H DOSY NMR spectroscopy

^1H DOSY NMR spectra were recorded on a Bruker Ascend 400 spectrometer. Chemical shifts are given in parts per million (ppm) and are referenced to the signals of the respective solvent. Either 4 mg of **L1** or 10 mg of cage **C1** were dissolved in a mixture of 500 µL DMF-d_7/D_2O (90-10) with the addition of either 2.50 mg (2 equiv.) or 3.74 mg (3 equiv.) of cisplatin and mixed properly. Afterward, a ^1H DOSY NMR was recorded for each experiment. Spectra were processed by MestreNova software.

2.2 ^{1}H NMR spectroscopy

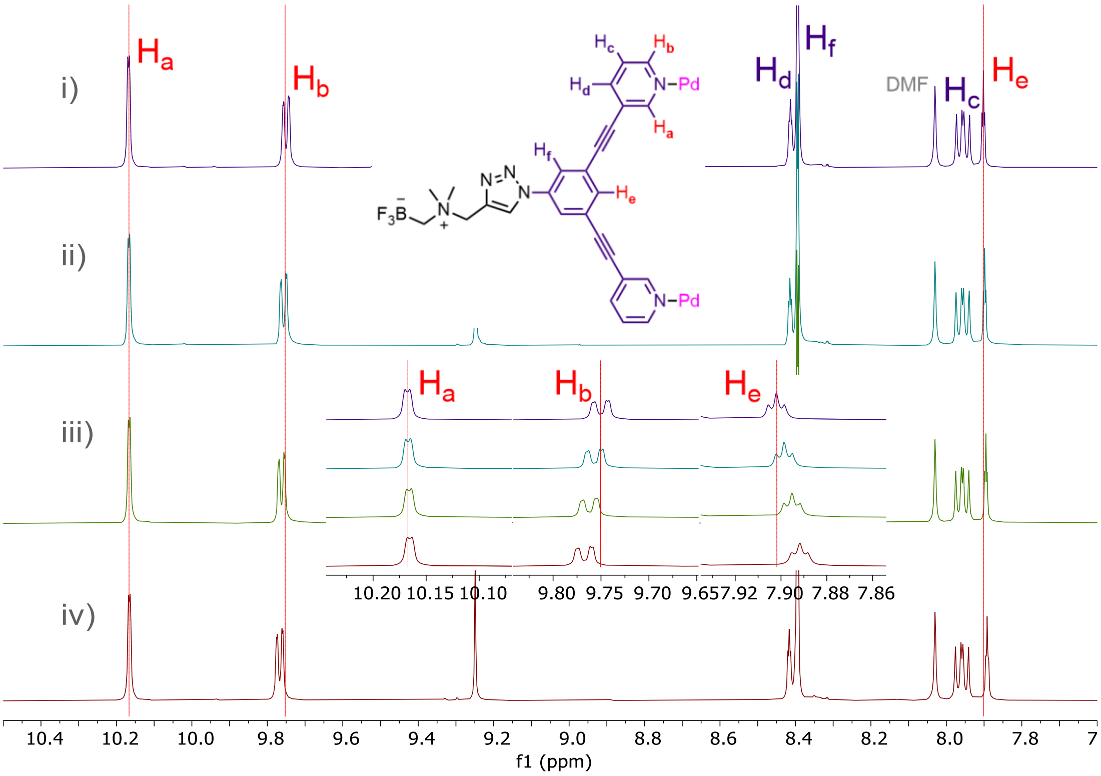

Figure S10. ^{1}H NMR of the cisplatin encapsulation event with **C1** in DMF-d_7. i) Empty cage. ii) Cage with 1 equiv. of cisplatin. iii) Cage with 2 equiv. of cisplatin. iv) Cage with 3 equiv. of cisplatin.

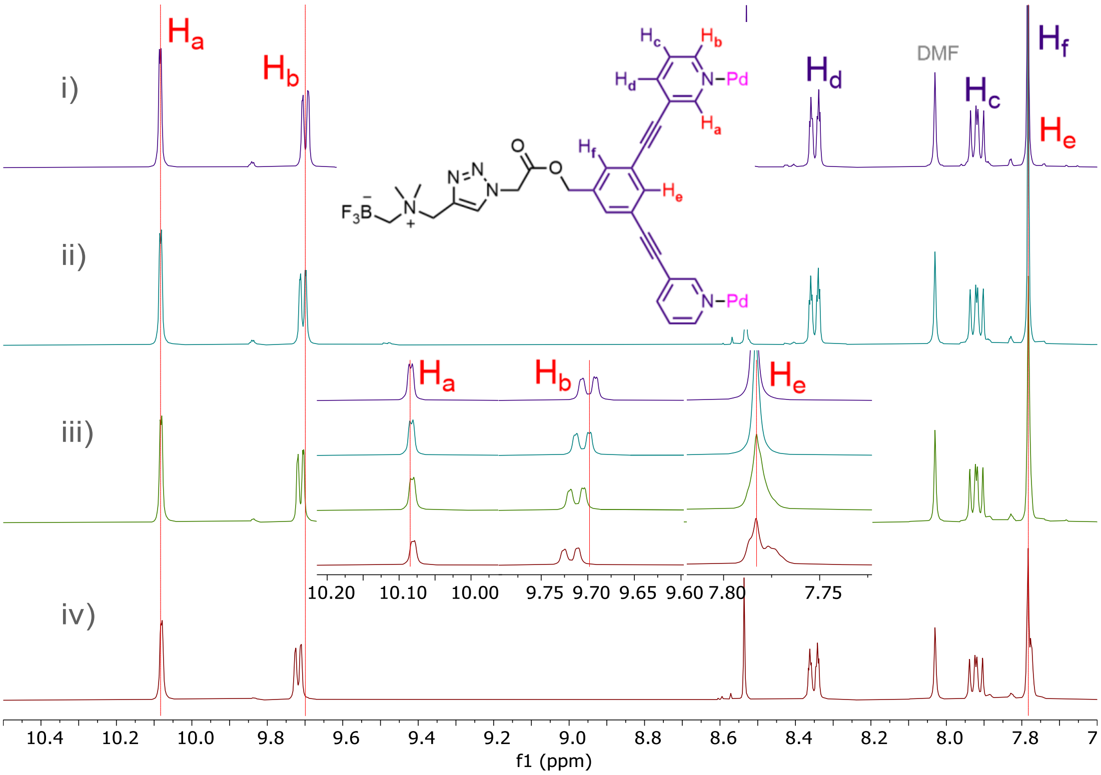

Figure S11. ^{1}H NMR of cisplatin encapsulation event with **C2** in DMF-d_7. i) Empty Cage. ii) Cage with 1 equiv. of cisplatin. iii) Cage with 2 equiv. of cisplatin. iv) Cage with 3 equiv. of cisplatin.

2.3 ^{195}Pt NMR spectroscopy

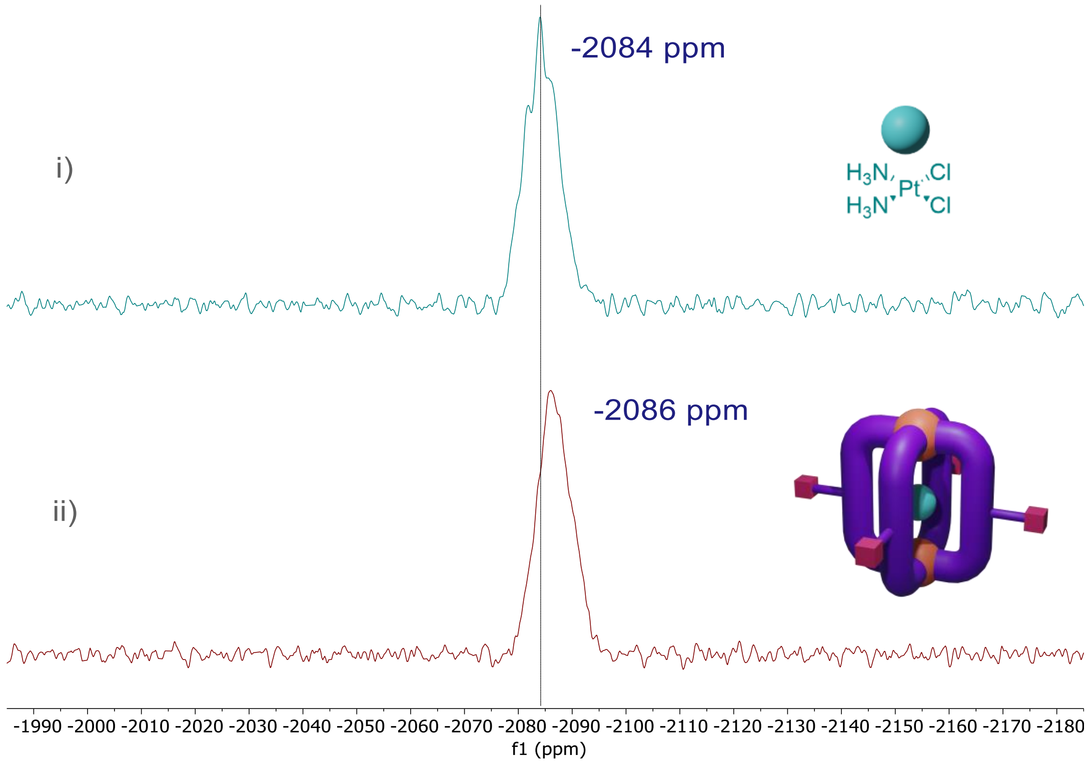

Figure S12. ^{195}Pt NMR study of cisplatin encapsulation with **C1** in DMF-d_7. i) Spectrum of free cisplatin; ii) spectrum of cage **C1** with 2 equiv. of cisplatin.

2.4 High resolution ESI mass spectrometry of cisplatin loaded C1

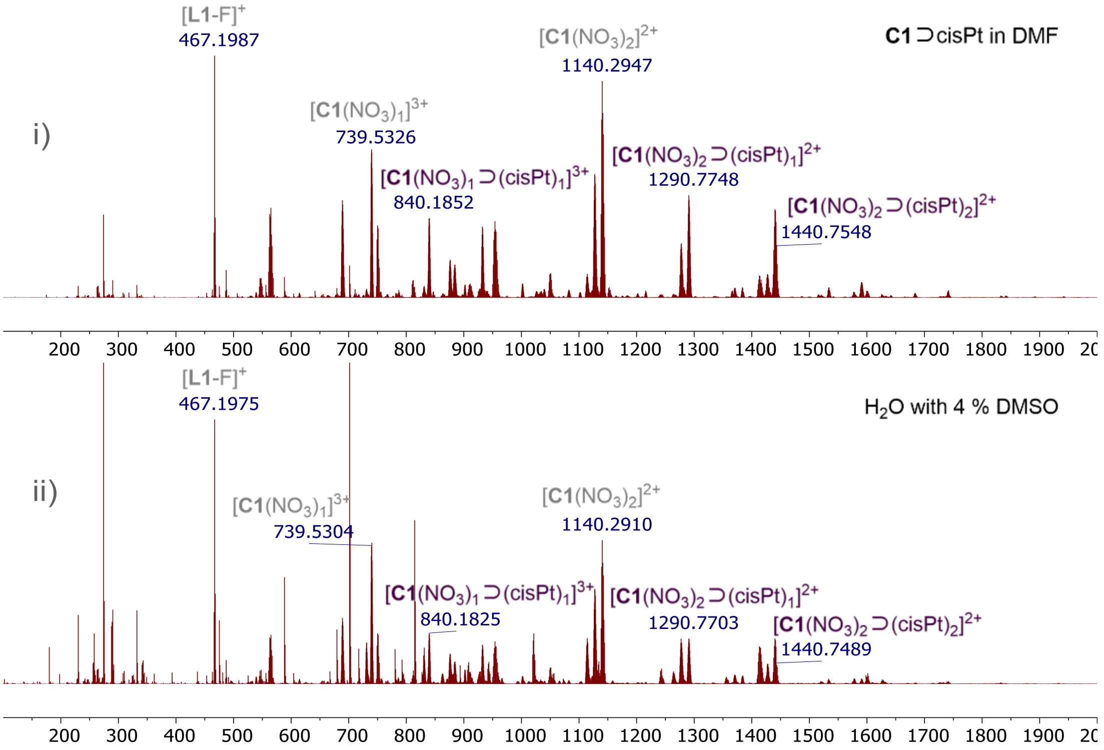

Figure S13a. High-resolution mass spectrum of **C1**⊃cisplatin. i) In DMF (direct inject., + mode, 80 dp). ii) In H_2O with 4% DMSO at 0.15 mM (direct inject., + mode, 130 dp).

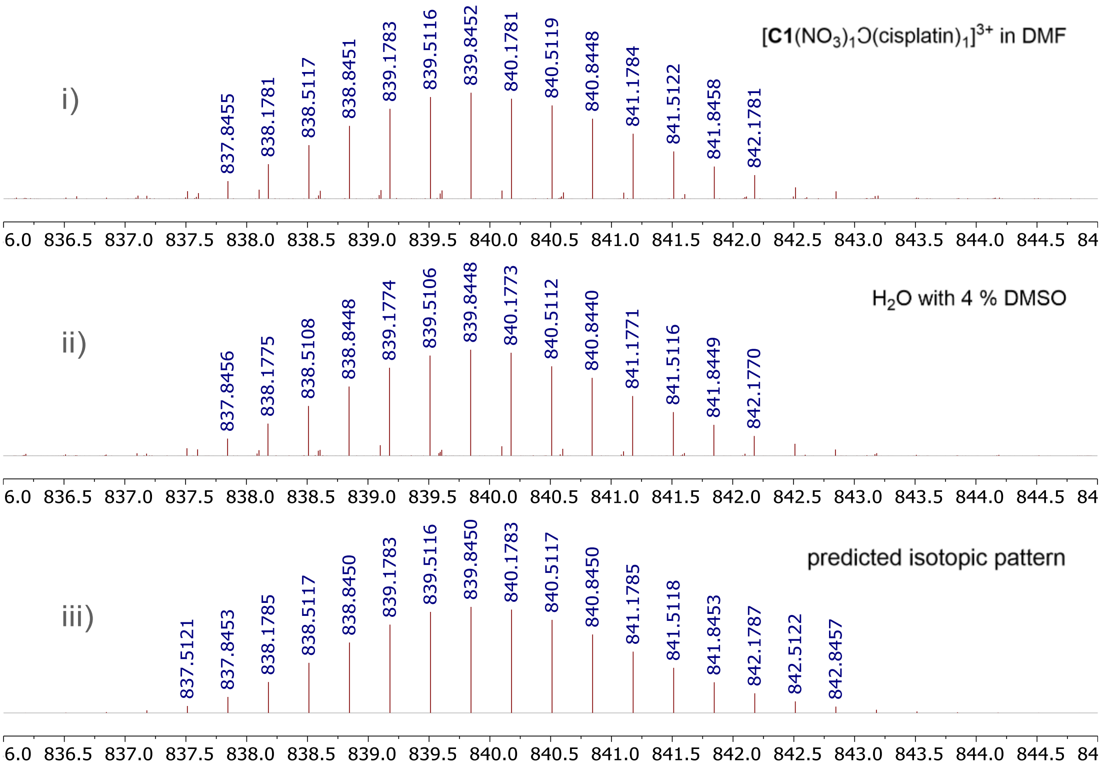

Figure S13b. Isotope patterns for [**C1**(NO_3)$_1$ ⊃(cisplatin)$_1$]$^{3+}$ observed by high-resolution mass spectrometry. i) In DMF (direct inject., + mode, 80 dp). ii) In H_2O with 4% DMSO at 0.15 mM (direct inject., + mode, 80 dp). iii) Predicted isotopic pattern.

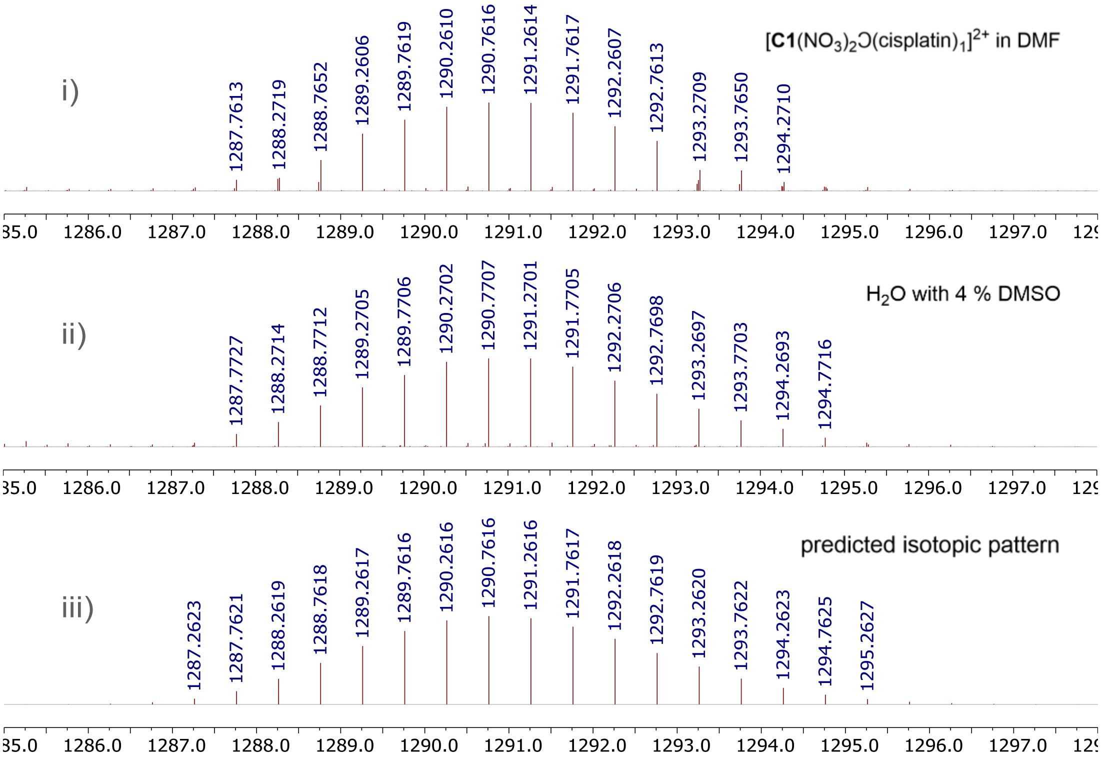

Figure S13c. Isotope patterns for [**C1**(NO_3)$_2$ ⊃(cisplatin)$_1$]$^{2+}$ observed by high-resolution mass spectrometry. i) In DMF (direct inject., + mode, 100 dp). ii) In H_2O with 4% DMSO at 0.15 mM (direct inject., + mode, 130 dp). iii) Predicted isotopic pattern.

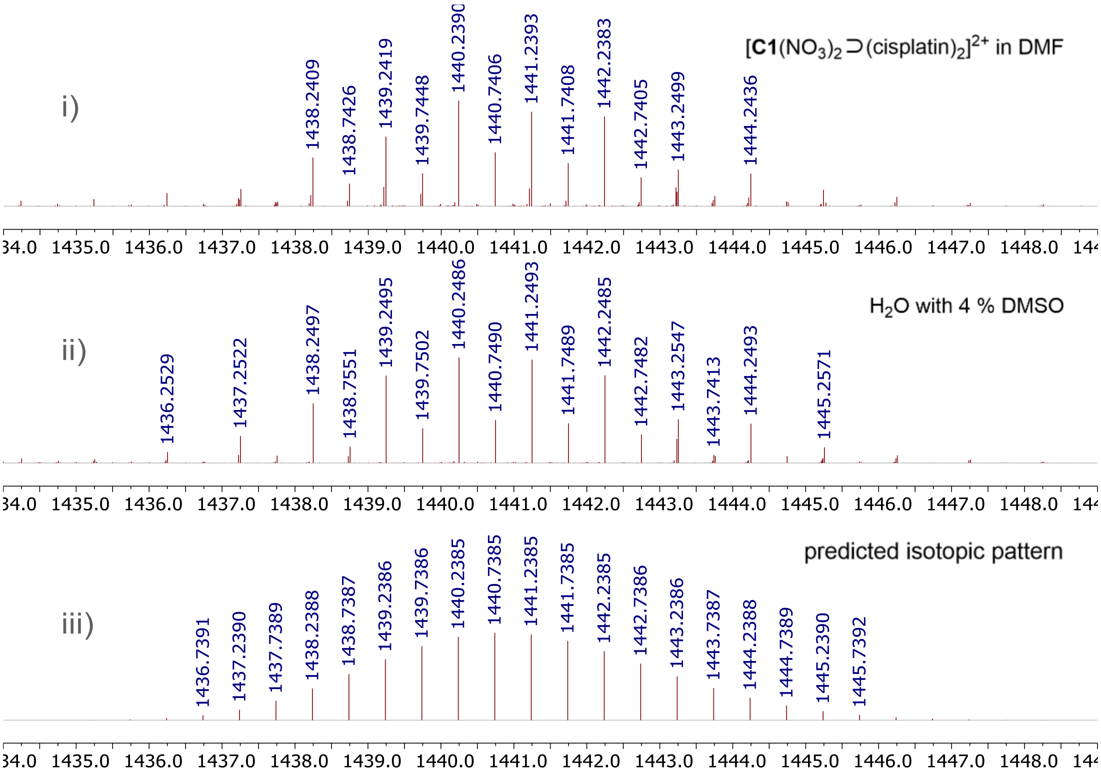

Figure S13d. Isotope patterns for [**C1**$(NO_3)_2$ ⊃(cisplatin)$_2$]$^{2+}$ observed by high-resolution mass spectrometry. i) In DMF (direct inject., + mode, 80 dp). ii) In H_2O with 4% DMSO at 0.15 mM (direct inject., + mode, 130 dp). iii) Predicted isotopic pattern.

2.5 High resolution ESI mass spectrometry of cisplatin loaded C2

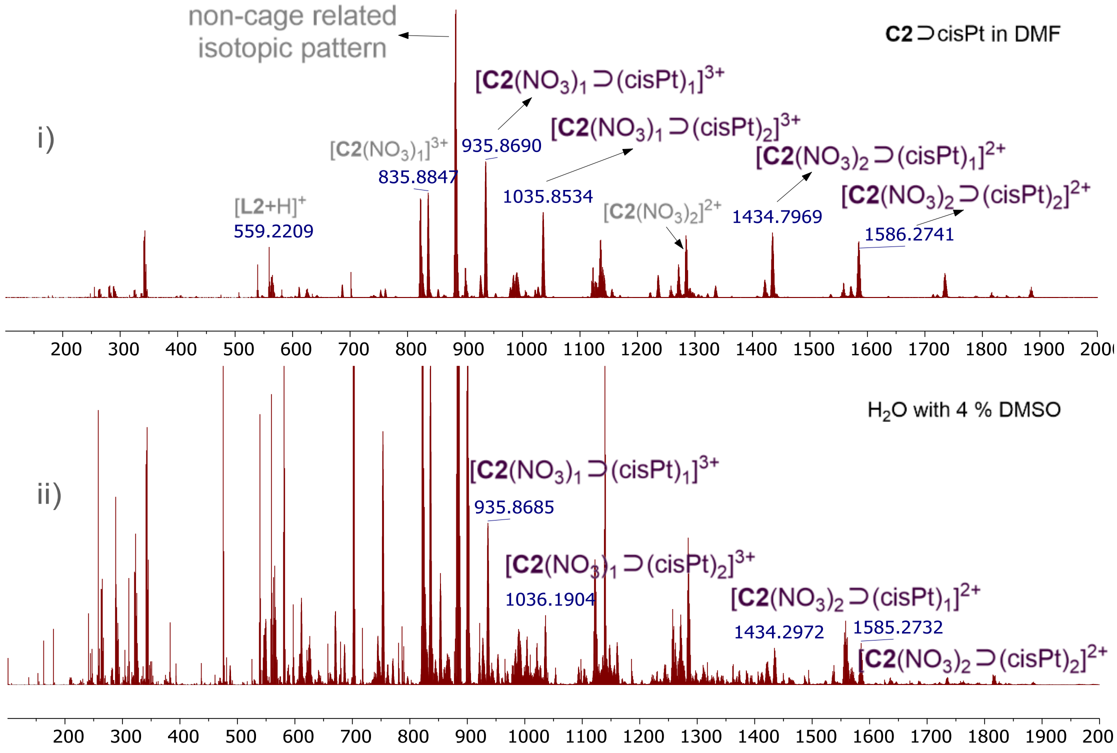

Figure S14a. High-resolution mass spectrum of **C2**⊃cisplatin. i) In DMF (direct inject., + mode, 80 dp). ii) In H_2O with 4% DMSO at 0.15 mM (direct inject., + mode, 80 dp).

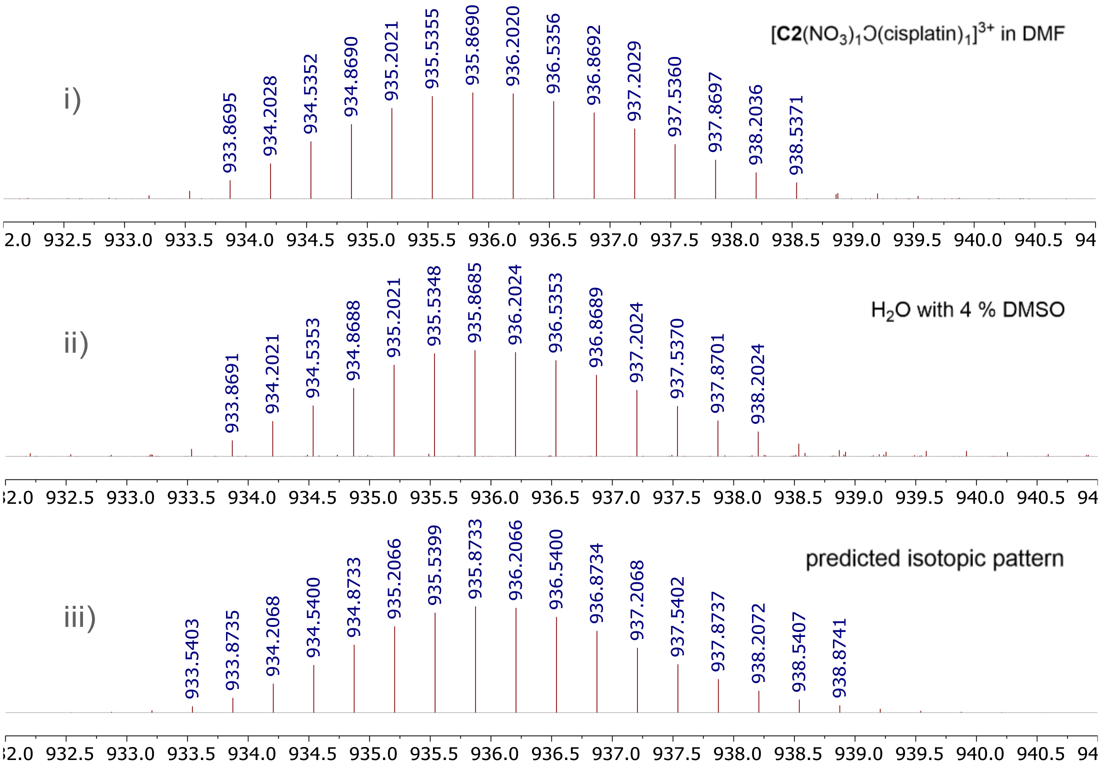

Figure S14b. Isotope patterns for [**C2**$(NO_3)_1$⊃(cisplatin)$_1]^{3+}$ observed by high-resolution mass spectrometry. i) In DMF (direct inject., + mode, 80 dp). ii) In H_2O with 4% DMSO at 0.15 mM (direct inject., + mode, 80 dp). iii) Predicted isotopic pattern.

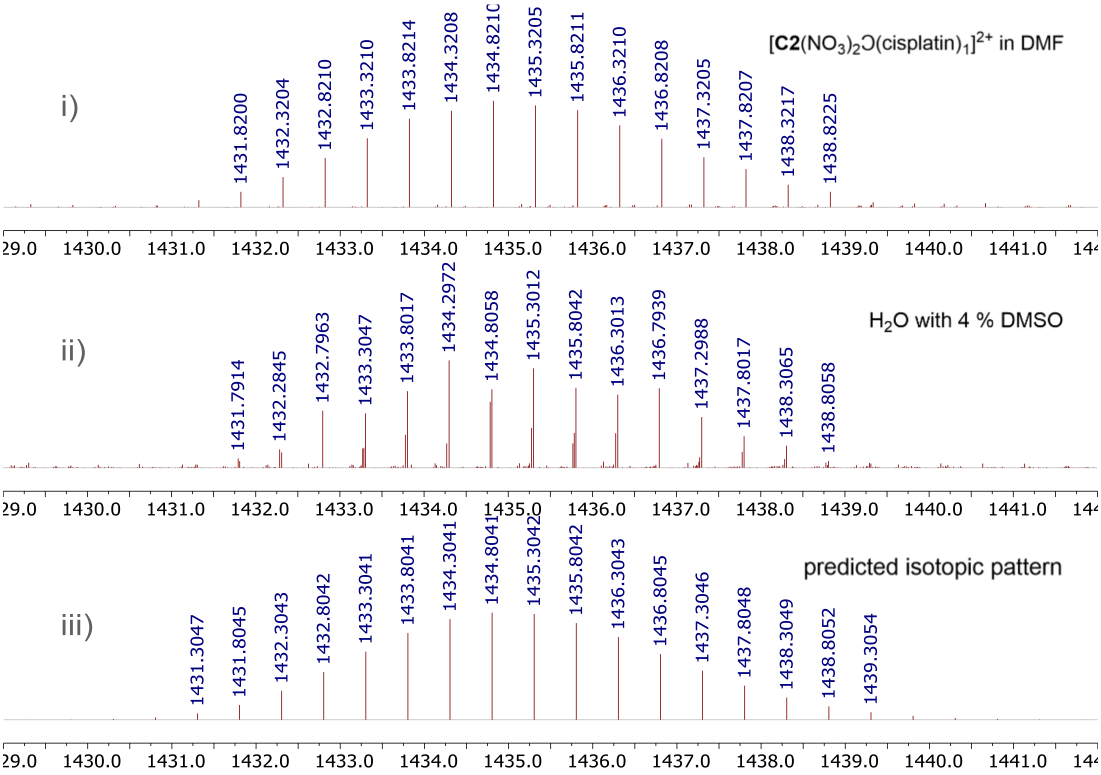

Figure S14c. Isotope patterns for [**C2**$(NO_3)_2$⊃(cisplatin)$_1]^{2+}$ observed by high-resolution mass spectrometry. i) In DMF (direct inject., + mode, 130 dp). ii) In H_2O with 4% DMSO at 0.15 mM (direct inject., + mode, 80 dp). iii) Predicted isotopic pattern.

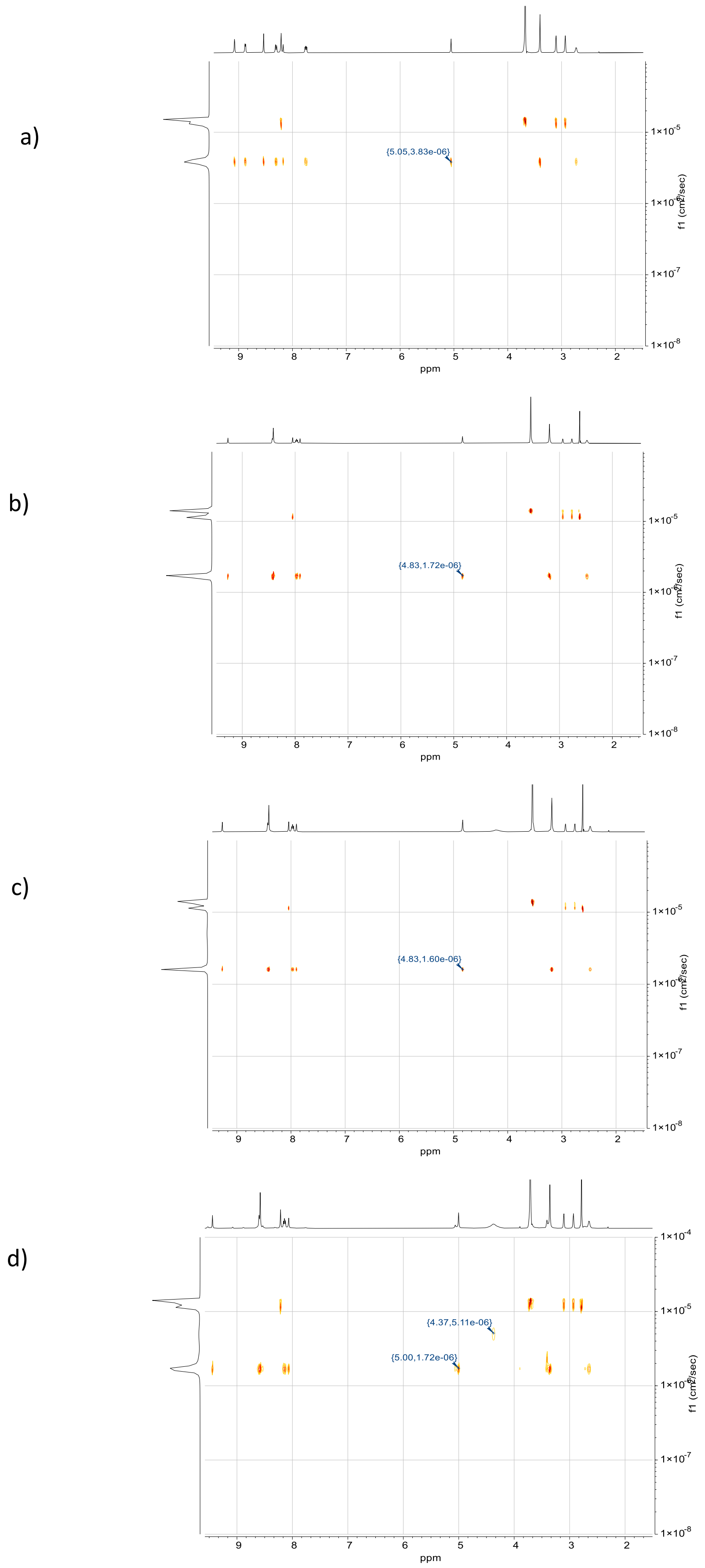

Figure S15. ^{1}H DOSY spectra (400 MHz) in DMF-d_7/D_2O *90:10*: a) ligand **L1**, b) **C1** cage, c) **C1** cage + 2 eq. Cisplatin, d) **C1** cage + 3 eq. Cisplatin.

3. Stability experiments

3.1 ^{1}H NMR spectroscopy

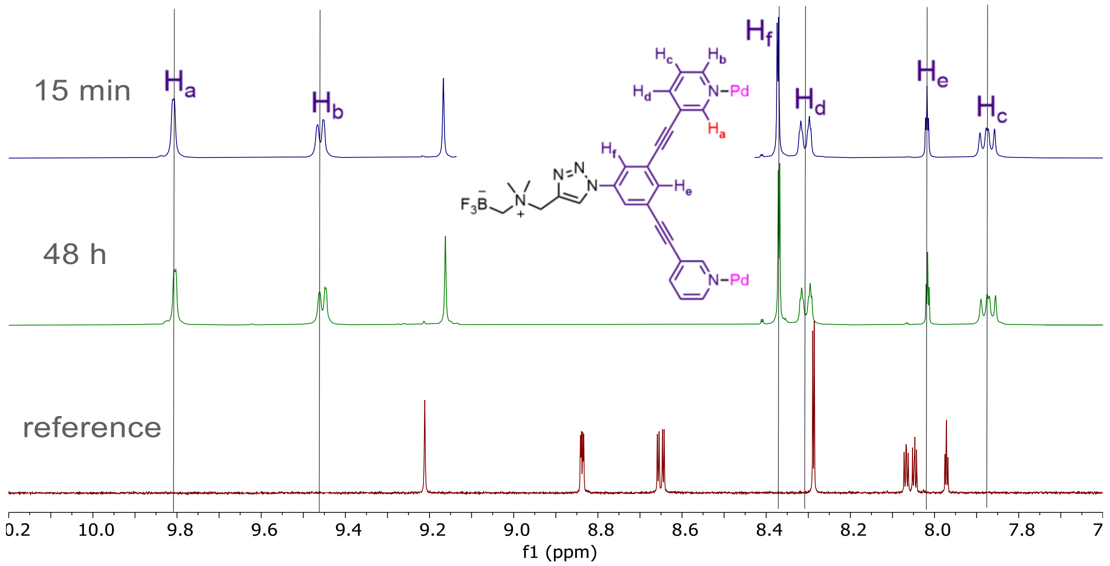

Figure S16. ^{1}H NMR of the empty cage **C1** in DMSO-d_6 showing the stability over time. i) Spectra recorded at 15 min after sample preparation. ii) Spectra recorded after 48 h. iii) Ligand **L1** as reference.

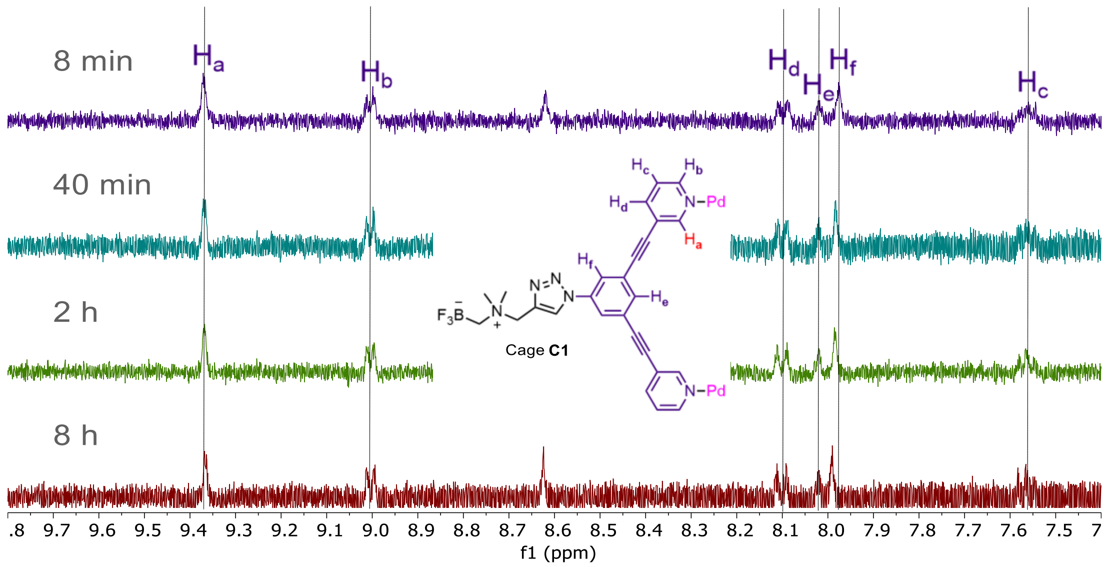

Figure S17. ^{1}H NMR of the empty cage **C1** in a mixture of 4 % DMSO-d_6 in D_2O showing the stability at different times. The concentration of the cage in the experiment was 1.5 mM, which is a somehow practicable concentration for NMR experiments.

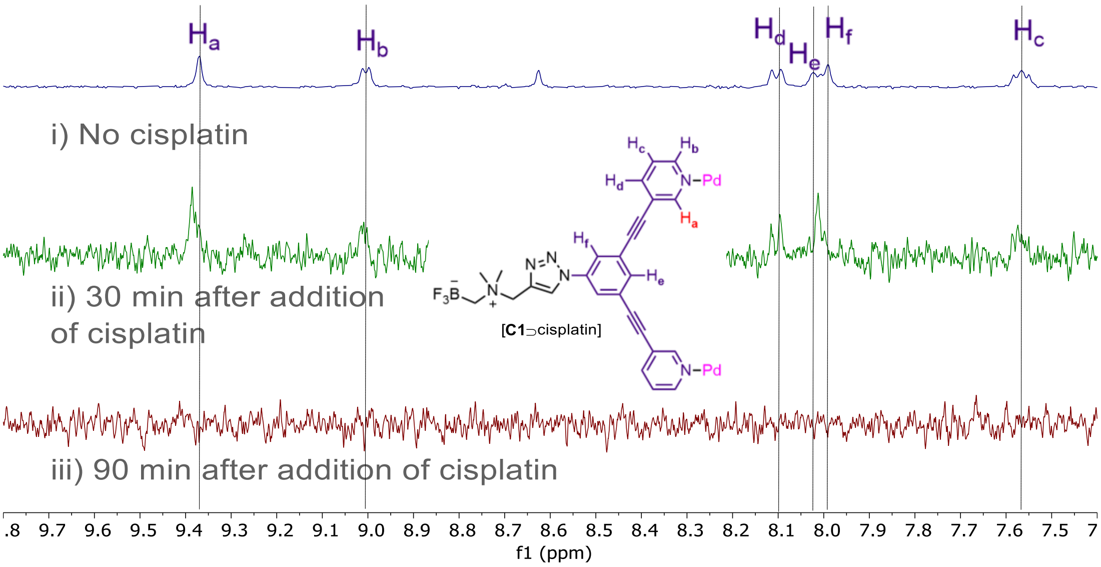

Figure S18. ^{1}H NMR of [**C1**⊃cisplatin] in a mixture of 4 % DMSO-d_6 in D_2O showing the stability at different times. The concentration of the cage in the experiment was 1.5 mM, which is a somehow practicable concentration for NMR experiments. i) Spectra recorded before the addition of cisplatin. ii) Spectra recorded 30 min after the addition of cisplatin. iii) Spectra recorded 90 min after the addition of cisplatin.

3.2 High-resolution ESI mass spectrometry

Mass spectrum of **C1** in a mixture of 4 % DMSO in H_2O at injection concentrations (0.15 mM).

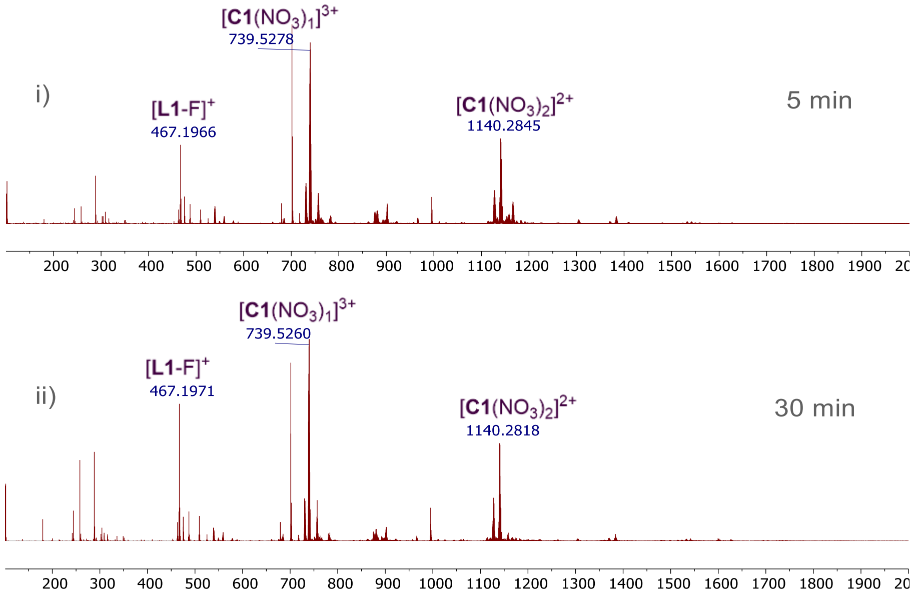

Figure S19. High-resolution mass spectrum of **C1** in a mixture of 4 % DMSO in H_2O at injection concentration (0.15 mM) showing the stability over time (direct inject., + mode, 80 dp). i) Spectrum recorded after sample preparation (t < 5 min). ii) Spectrum of the same sample recorded after 30 min of incubation.

Zoom of **C1** in mixtures of 4 % DMSO in saline at injection concentrations (0.15 mM).

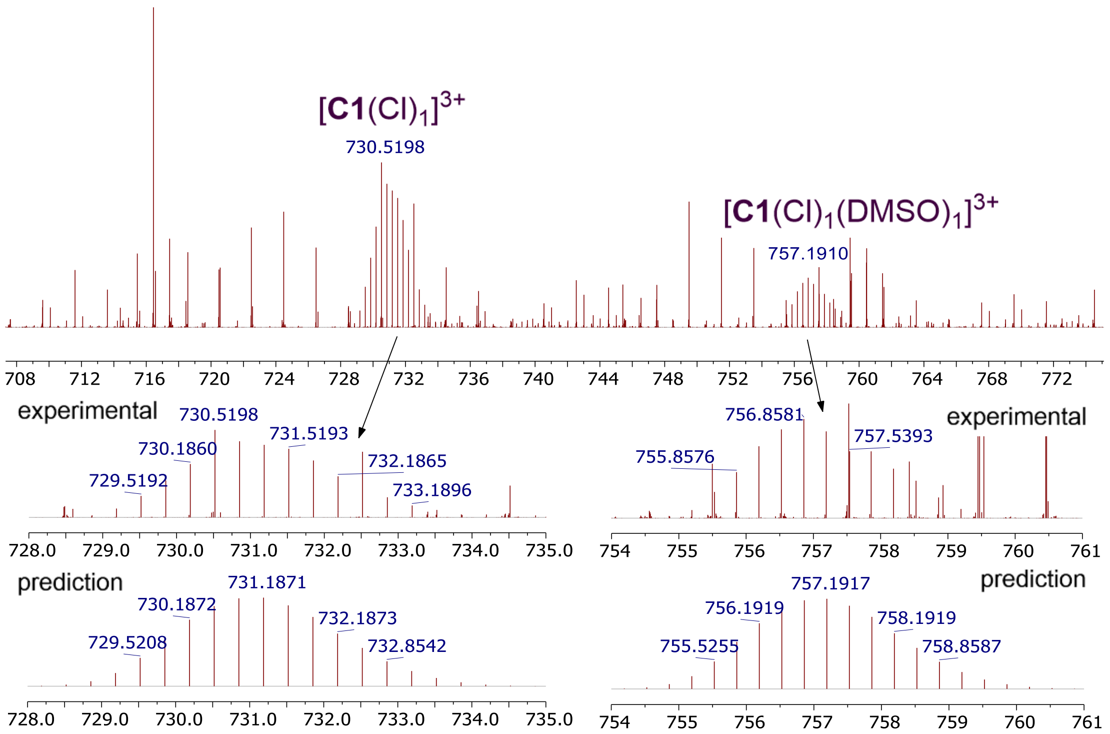

Figure S20a. Zoom between 650-850 Da of the high-resolution mass spectrum of **C1** in a mixture of 4 % DMSO in saline (0.045 % NaCl) at injection concentration (0.15 mM), showing the cage ionized with chlorine ions [**C1**$(Cl)_1]^{3+}$ and [**C1**$(Cl)_1(DMSO)_1]^{3+}$.

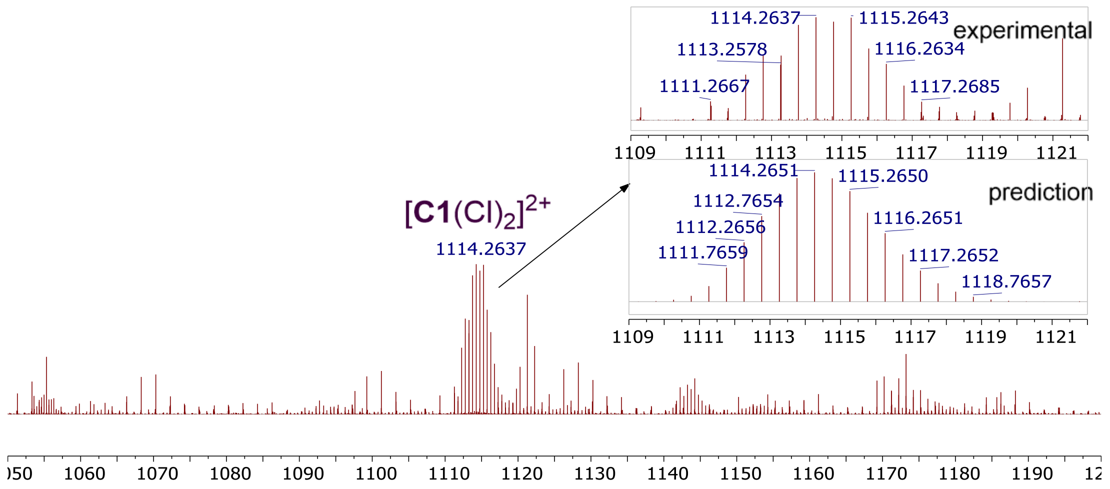

Figure S20b. Zoom between 1050-1200 Da of the high-resolution mass spectrum of **C1** in a mixture of 4 % DMSO in saline (0.045 % NaCl) at injection concentration (0.15 mM), showing the cage ionized with chlorine ions [**C1**$(Cl)_2]^{2+}$.

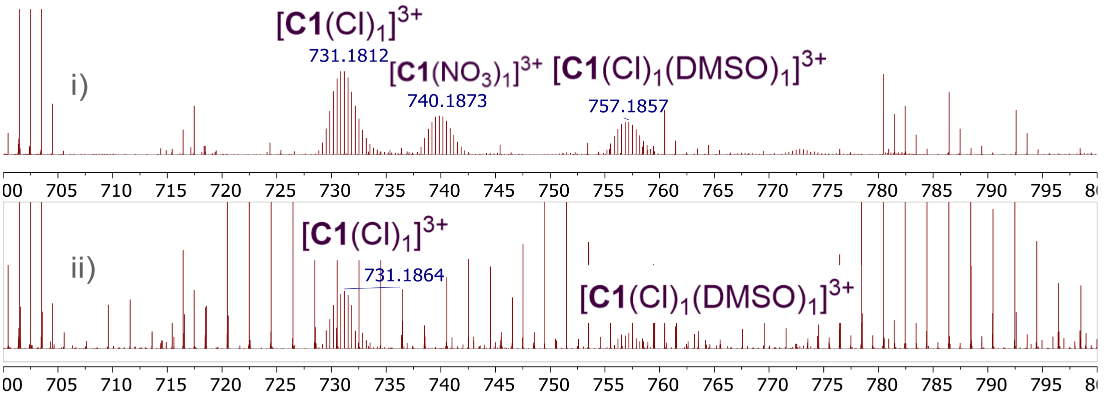

Figure S20c. Zoom between 600-800 Da of the high-resolution mass spectrum of **C1**. i) In a mixture of 4 % DMSO in saline (0.0036 % NaCl) at injection concentration (0.15 mM), in which this lower concentration of NaCl still allows to visualize the [**C1**$(NO_3)_1]^{3+}$ ion. ii) In a mixture of 4 % DMSO in saline (0.09 % NaCl) at injection concentration (0.15 mM), which is the most similar concentration to *in vivo* conditions (0.9 % NaCl) where the ionized cage can be visualized.

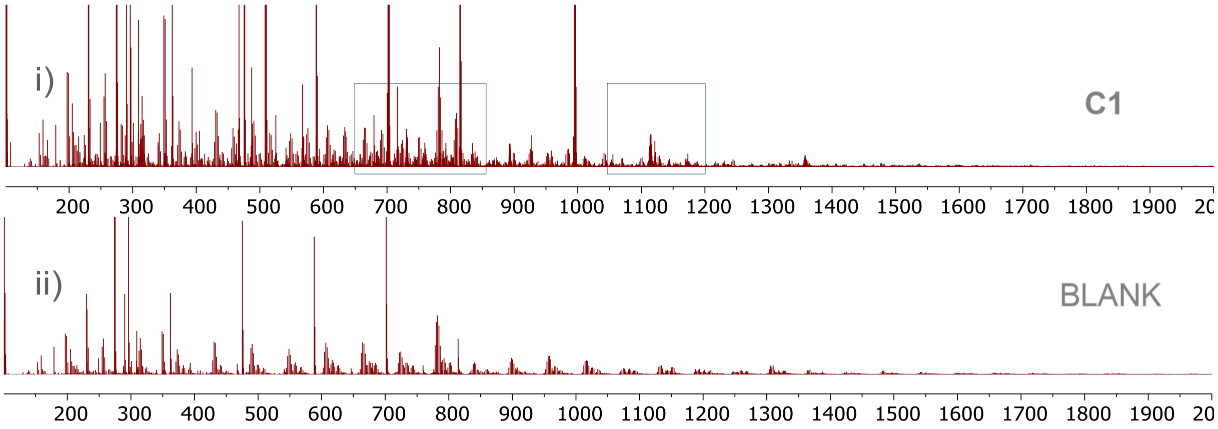

Figure S20d. i) High-resolution mass spectrum of **C1** in a mixture of 4 % DMSO in saline (0.045 % NaCl) at injection concentration (0.15 mM) showing the high complexity spectrum because of the clustering effect of the presence of NaCl. Relevant zones showed earlier in figures S18a-c has been highlighted. ii) Blank of a solution of 4 % DMSO in saline (0.045 % NaCl). Direct inject., + mode, 80 dp.

4. Radiolabelling and biodistribution studies

4.1 Radiochemistry

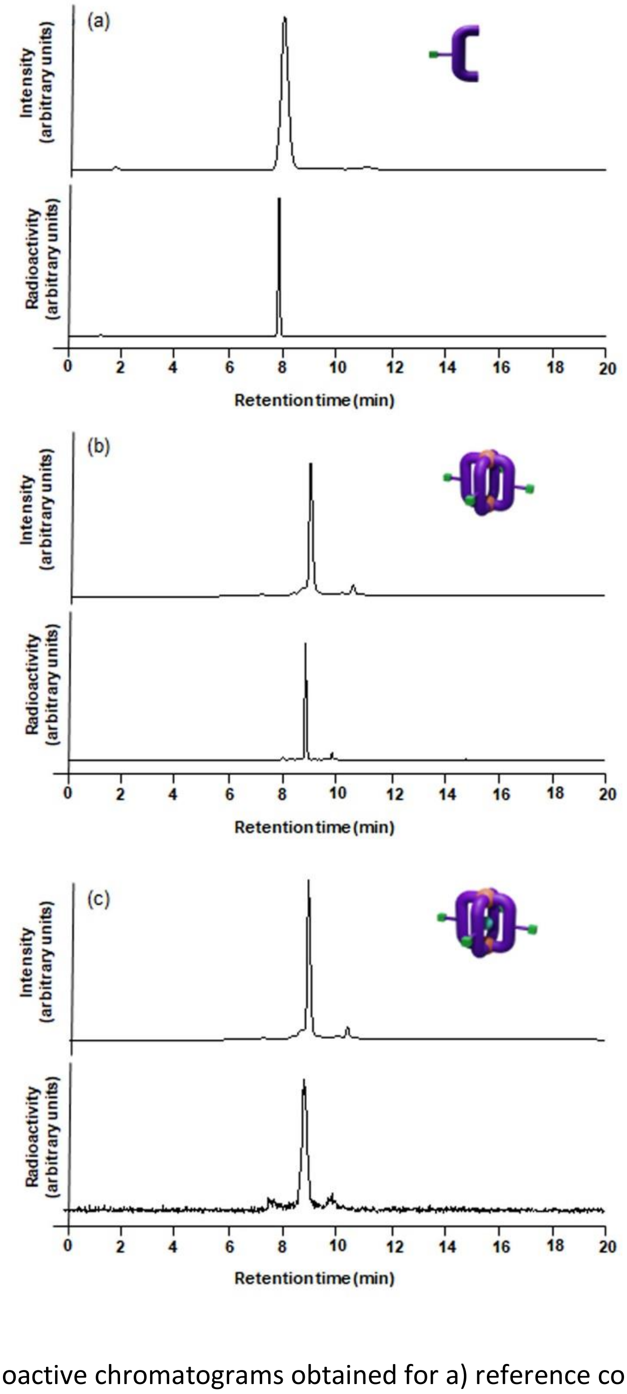

Figure S21. UV and radioactive chromatograms obtained for a) reference compound **L1** (top) and **^{18}F-L1** (bottom), b) reference compound **C1** (top) and **^{18}F-C1** (bottom), and c) reference compound **[C1(NO_3)$_4$⊃cisplatin]** (top) and **^{18}F-[C1(NO_3)$_4$⊃cisplatin]** (bottom).

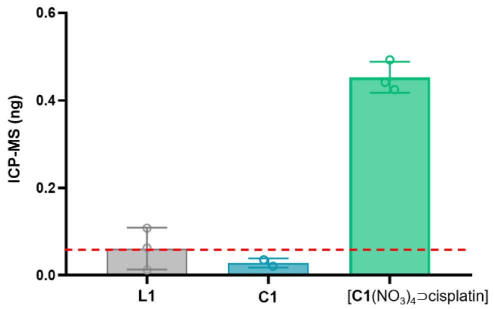

Figure S22. Amount of Pt detected in **L1**, **C1** and [**C1**$(NO_3)_4$⊃cisplatin] after manual collection of chromatographic peaks, as determined by ICP-MS (background indicated by red line).

4.2 *In vivo* and *Ex vivo* studies

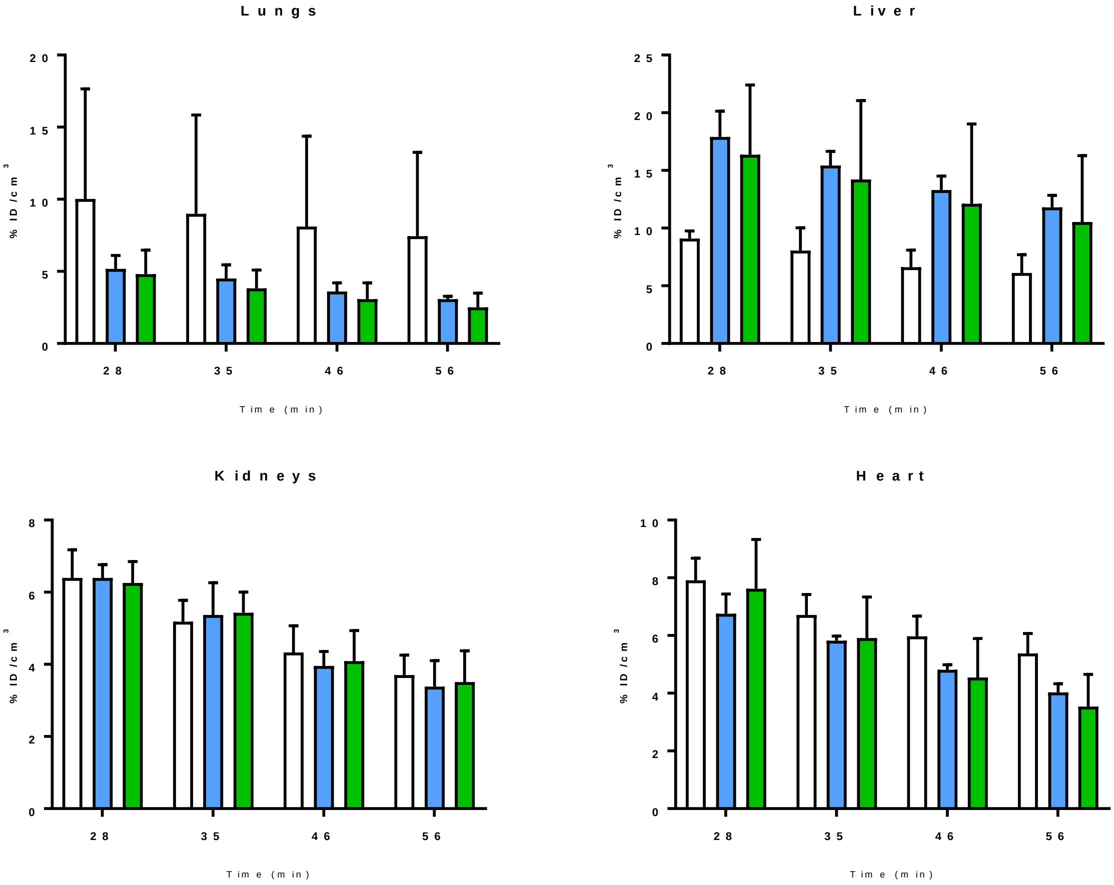

Figure S23. Concentration of radioactivity, expressed as percentage of injected dose per cubic centimeter of tissue, obtained for the different organs at 28, 35, 46 and 56 min after administration of of ^{18}F-**L1** (white), ^{18}F-**C1** (blue) and ^{18}F-[**C1**$(NO_3)_4$⊃cisplatin] (green).
